# Toxic aldehyde generation in and food uptake from culinary oils during frying practices: peroxidative resistance of a monounsaturate-rich algae oil

**DOI:** 10.1038/s41598-019-39767-1

**Published:** 2019-03-11

**Authors:** Sarah Moumtaz, Benita C. Percival, Devki Parmar, Kerry L. Grootveld, Pim Jansson, Martin Grootveld

**Affiliations:** Leicester School of Pharmacy, De Montfort University, The Gateway, Leicester, LE1 9BH United Kingdom

## Abstract

Human ingestion of cytotoxic and genotoxic aldehydes potentially induces deleterious health effects, and high concentrations of these secondary lipid oxidation products (LOPs) are generated in polyunsaturated fatty acid (PUFA)-rich culinary oils during high temperature frying practices. Here, we explored the peroxidative resistance of a novel monounsaturate-rich algae frying oil (MRAFO) during laboratory-simulated shallow- and domestically-based repetitive deep-frying episodes (LSSFEs and DBRDFEs respectively), the latter featuring potato chip fryings. Culinary frying oils underwent LSSFEs at 180 °C, and DBRDFEs at 170 °C: aldehydes were determined by ^1^H NMR analysis in samples collected at increasing heating/frying time-points. Fast food restaurant-fried potato chip serving (FFRPCS) aldehyde contents were also monitored. Substantially lower levels of aldehydes were generated in the MRAFO product than those observed in PUFA-richer oils during LSSFEs. Toxicologically-significant concentrations of aldehydes were detected in FFRPCSs, and potato chips exposed to DBRDFEs when using a PUFA-laden sunflower oil frying medium: these contents increased with augmented deep-frying episode repetition. FFRPCS aldehyde contents were 10–25 ppm for each class monitored. In conclusion, the MRAFO product generated markedly lower levels of food-penetrative, toxic aldehydes than PUFA-rich ones during LSSFEs. Since FFRPCS and DBRDFE potato chip aldehydes are predominantly frying oil-derived, PUFA-deplete MRAFOs potentially offer health-friendly advantages.

## Introduction

The peroxidation of unsaturated fatty acids (UFAs) at temperatures commonly used for standard frying or cooking episodes (*ca*. 180 °C) is an extremely complex chemical degradation process which involves highly-reactive free radical species, and/or alternatively, singlet oxygen (^1^O_2_). Mechanisms available for this process primarily involve the oxidative conversion of such UFAs to primary lipid oxidation products (LOPs), commonly described as lipid hydroperoxides (also known as hydroperoxymonoenes and conjugated hydroperoxydienes according to their fatty acid (FA) sources, abbreviated HPMs and CHPDs respectively), a process sequentially followed by their fragmentation to secondary ones, the latter including extremely toxic aldehydes in particular^1,2^. Further HPM and CHPD degradation products include epoxy acids, alcohols, ketones, oxoacids, alkanes and alkenes, in addition to further toxic oxidation and fragmentation products^1–5^.

However, HPM-generating monounsaturated fatty acids (MUFAs) are much more resistant to oxidation than polyunsaturated ones (PUFAs), and hence they give rise to lower levels of only particular LOPs when heated in this manner, and generally only after exposure to prolonged thermal stressing episodes at standard frying temperatures. Therefore, the order and extent of toxic LOP production in culinary oils is PUFAs > MUFAs >>> saturated fatty acids (SFAs), and the relative oxidative susceptibilities of 18-carbon chain length fatty acids (FAs) containing 0, 1, 2 and 3 carbon-carbon double bonds (i.e. >C=C< functions) are 1:100:1,200:2,500 respectively^4^. Moreover, the rate of fragmentation of lipid hydroperoxides to the above series of low-molecular-mass degradation products also increases with increasing FA unsaturation status, i.e. it is in the order linolenoyl- >linoleoyl-≫ oleoylglycerols^4,5^.

Previous NMR-based investigations focused on the peroxidative degradation of culinary oil UFAs during standard frying practices, or corresponding laboratory-simulated thermal stressing episodes, have demonstrated the thermally-promoted generation of very high levels of highly toxic aldehydes and their hydroperoxide precursors in such products (particularly those rich in PUFAs)^6–8^, and these results have been available to the scientific, food and public health research communities since 1994^6^. Indeed, samples of repeatedly-used oils collected from domestic kitchens, fast-food retail outlets and restaurants, have confirmed the generation of these aldehydes at high concentrations during ‘on-site’ frying practices. Such results have been repeated, replicated and ratified by many other research laboratories worldwide (most notably^9^). We can also employ these NMR techniques to monitor the corresponding degradation of culinary oil PUFAs and MUFAs during such standard frying/cooking practices^6,7^, and also to detect and quantify a range of further LOPS, i.e. CHPDs and HPMs, ketones and alcohols, together with toxic epoxy acids, the latter including leukotoxin and its derivatives such as isoleukotoxin and leukotoxindiol^6–9^.

Of critical importance to their public health risks as food-borne toxins, typical chemically-reactive α,β-unsaturated aldehydes produced during the thermal stressing of culinary oils according to standard frying practices are absorbed from the gut into the systemic circulation *in vivo*^10^ following oral ingestion, where they have access and may cause damage to cells, tissues and essential organs. Indeed, these agents have been demonstrated to promote a broad spectrum of concentration-dependent cellular stresses, and their adverse health properties include effects on critical metabolic pathways (for example, energy metabolism^11^); the promotion and perpetuation of atherosclerosis and cardiovascular diseases^10,12–14^; mutagenic and carcinogenic properties^15–19^; teratogenic actions (embryo malformations during pregnancy^20^); the exertion of striking pro-inflammatory effects^21,22^; the induction of gastropathic properties (peptic ulcers) following dietary ingestion^23^; neurotoxic actions, particularly for 4-hydroxy-*trans*-2-nonenal (HNE) and -hexenal (HHE)^24^; and impaired vasorelaxation coupled with the adverse stimulation of significant increases in systolic blood pressure^25^. Further deleterious health effects include chromosomal aberrations, which are reflective of their clastogenic potential, sister chromatid exchanges and point mutations, in addition to cell damage and death^26,27^.

The toxicity of these aldehydes, particularly the α,β-unsaturated ones, is ascribable to their aggressive chemical reactivity. Indeed, they cause damage to critical biomolecules such as DNA: since they are powerful electrophilic alkylating agents, α,β-unsaturated aldehydes readily alkylate DNA base adducts, and this serves to explain their mutagenic and carcinogenic properties^16–18^. Moreover, these secondary LOPs are also able to form covalent adducts with many proteins via Schiff base or Michael addition reactions^7,10^, and these can induce significant structural and conformational changes in these macromolecules, which serve to impair their biocatalytic functions. However, until recently, such toxicological considerations have generally continued to elude interest or focus from many food industry and public health researchers.

The recent development of algae-derived cooking oils has provided much scope and benefits regarding the effective bioengineering of their triacylglycerol FA contents, in particular for their valuable uses as cooking oils. Indeed, the novel MUFA-rich algae frying oil (MRAFO) tested in this work (Thrive™ high stability culinary algae oil, TerraVia Holdings Inc., South San Francisco, CA, USA) represents the first ever such algal product available to consumers in the USA. In addition to its potential resistance to thermally-induced, O_2_-fueled peroxidation during common frying cycles, this predominantly MUFA-containing [i.e. >90% (w/w)] culinary oil offers further major advantages, including a very high smoke-point of 252 °C (which is significantly greater than those of alternative cooking oils such as sunflower, corn, canola, olive and groundnut oils), and also a neutral taste contribution.

Therefore, in view of the much lowered susceptibility of MUFAs to oxidation than PUFAs, in this study we have explored the oxidative resistance of the above high-stability MRAFO product during laboratory-simulated standard shallow frying practices, i.e. one of its major culinary applications, and also during DBRDFEs. For this purpose, we exposed this MRAFO, in addition to commonly-utilised sunflower, corn, canola and extra-virgin olive oils, to these episodes at 180 °C and 170 °C respectively, and have employed multicomponent high-resolution ^1^H NMR analysis to determine the concentrations of a series of highly toxic classes of aldehydic LOPs therein as a function of heating time, i.e. from 0–90 min. for LSSFEs, and 8 × 10 min. DBRDFEs (the latter featuring a 30 min. oil cooling rest period between each frying cycle). The time-dependent production of epoxy acid LOP toxins was simultaneously monitored in all oils investigated. Such experiments serve to provide valuable information and insights regarding the possible health-threatening effects of these aldehydes when ingested in human diets featuring fried food sources of these toxins, e.g. potato chips, fish fillets, battered chicken, chicken strips, etc., and here we have also demonstrated, for the first time, the availability for human consumption of high, toxicologically-significant (up to 25 ppm) levels of two major classes of α,β-unsaturated, and one major class of saturated aldehydes in servings of fried foods collected directly from fast food retail outlets/restaurants, and also in potato chips subjected to DBRDFEs when PUFA-rich sunflower oil is used as the frying medium. The potential deleterious health effects presented by these oils when employed as frying media, particularly those associated with PUFA-rich frying oil sources of dietary aldehydes, are discussed in detail.

## Results

### ^1^H NMR analysis and time-dependent monitoring of aldehydic LOPs in thermally-stressed culinary oils

**Figure 1:**
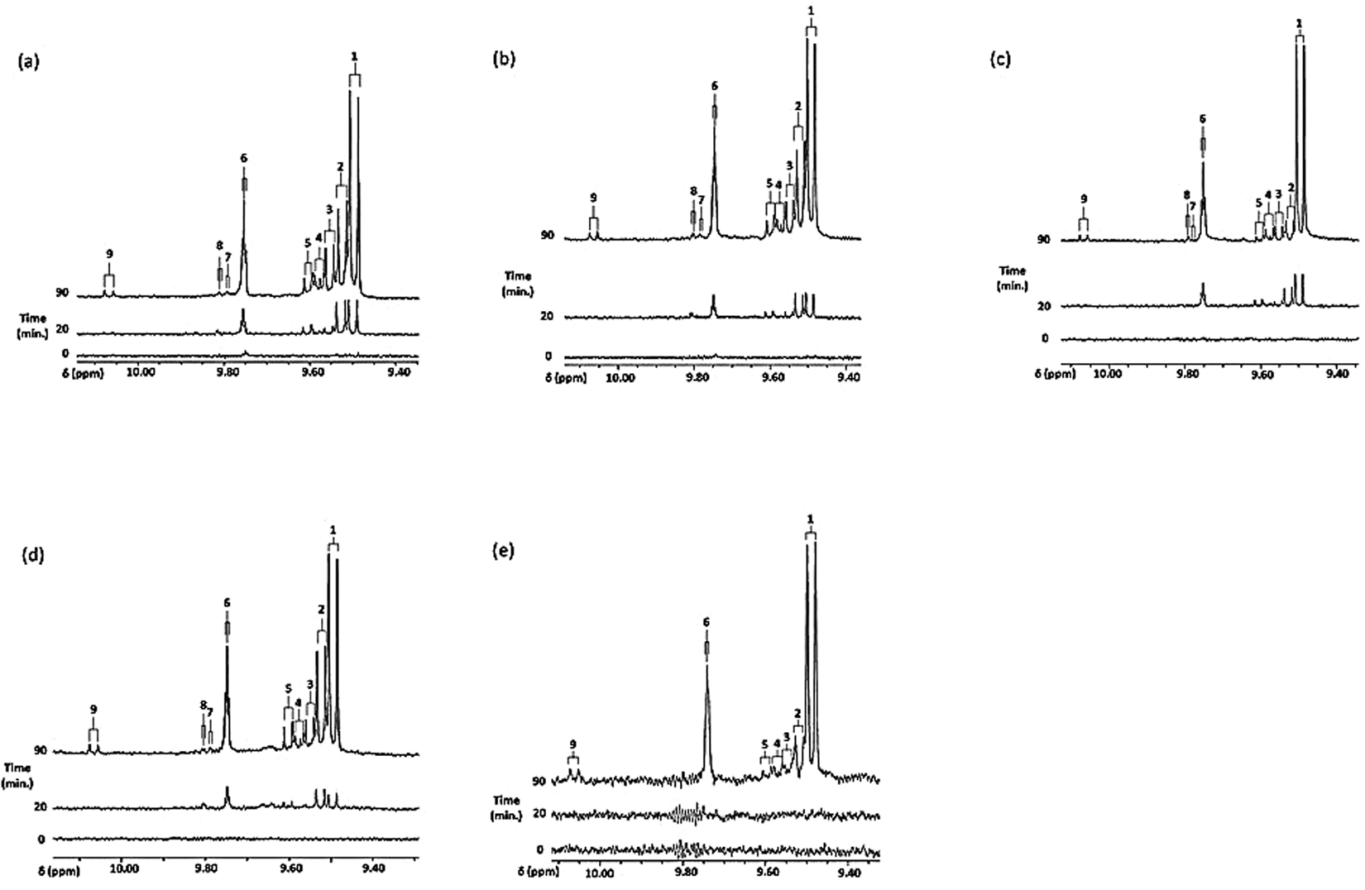
Expanded aldehydic-CHO proton (9.44–10.12 ppm) regions of the 400 MHz ^1^H NMR spectra of (**a**) sunflower, (**b**) corn, (**c**) extra-virgin olive, (**d**) canola, and (**e**) MRAFO oils exposed to thermal stressing episodes at 180 °C for periods of 0, 20 and 90 min. according to laboratory-simulated shallow frying practices (samples were collected for ^1^H NMR analysis at the 0, 5, 10, 20, 30, 60 and 90 min. time-points). Typical spectra are shown. Abbreviations: Number labels correspond to the -CHO function resonances of 1, *trans*-2-alkenals; 2, *trans*,*trans*-alka-2,4-dienals; 3, 4,5-epoxy-*trans*-2-alkenals; 4, combined 4-hydroxy/4-hydroperoxy-*trans*-2-alkenals; 5, *cis,trans*-alka-2,4-dienals; 6, *n*-alkanals; 7, 4-oxo-*trans*-2-alkenals; 8, low-molecular-mass short-chain *n*-alkanals, particularly propanal and *n*-butanal; and 9, *cis*-2-alkenals, a previously unidentified unsaturated aldehyde classification. All resonances visible are doublets, with the exception of 6 and 8, which are triplets (*j* = 1.69 and 1.78 Hz respectively). The intensity axis in **(e)** has been expanded for purposes of clarity.

^1^H NMR analysis demonstrated the thermally-induced generation of aldehydic LOPs in all oils investigated, and Fig. 1 shows partial ^1^H NMR profiles demonstrating the time-dependent production of -CHO function resonances ascribable to a range of these toxins when culinary oil products were heated according to our LSSFEs (assignments for these signals were confirmed via the acquisition of corresponding one- and two-dimensional ^1^H-^1^H COSY and TOCSY spectra for each heated oil, together with standard addition ‘spiking’ experiments performed with calibrated standard solutions of authentic aldehydes in C^2^HCl_3_). Spectra of heated sunflower, corn and canola oils also contained resonances assignable to aldehydic precursors, in particular *cis*,*trans*- *and trans,trans*-CHPDs (multiplet conjugated diene vinylic proton resonances located within the 5.40–6.60 and 5.40–6.30 ppm spectral regions respectively, together with broad -OOH function signals located at δ = 8.40–8.85 ppm), and *cis,trans*-conjugated hydroxydienes (δ = 5.40–6.50 ppm range), as previously reported^6–8^; broad -OOH resonances were also visible in spectra acquired on thermally-stressed extra-virgin olive oil and MRAFO products, although presumably they largely arise from HPMs rather than CHPDs in these cases. The CHPDs detectable are produced during recycling O_2_- and heat-stimulated peroxidative bursts throughout the whole simulated frying process, especially since these LOPs remain detectable in spectra acquired on the PUFA-rich oils after a 90 min. LSSFE period. Moreover, relatively low concentrations of these aldehydes and their CHPD precursors were also detectable in unheated sunflower and corn oil products [Fig. 1(a) and (b)].

As expected, absolute total, and total saturated and α,β-unsaturated aldehyde levels increased proportionately with oil PUFA content, and clearly >75% of these were of the more highly toxic α,β-unsaturated class. These included *trans*-2-alkenals [(*E)*-2-alkenals], *trans,trans-* and *cis,trans-*alka-2,4-dienals [(*E*,*E*)- and (*Z,E)*-2,4-alkadienals respectively], along with 4-hydroperoxy-/4-hydroxy-, and 4,5-epoxy-*trans*-2-alkenals [the latter three all substituted (*E)*-2-alkenal derivatives] (Fig. 1).

1D ^1^H-^1^H COSY and TOCSY NMR analysis of the 10.05–10.08 ppm ISB -CHO function doublet resonance (*j* = 8.1 Hz) confirmed that it was assignable to an α,β-unsaturated aldehyde, since it was directly connected to two vinylic proton multiplet signals located at δ = 6.59 and 5.95 ppm (Figure S1). This pattern of coupled resonances is consistent with that of *cis(Z)*-2-butenal (*cis*-crotonaldehyde), which has corresponding signals at δ = 10.10 ppm (*d, j* = 8.1 Hz), 6.70 (*dq*) and 5.97 ppm (*ddq*) [Figure S1(c)], and therefore this 10.05–10.08 ppm ISB resonance was assigned to *cis*-2-alkenals, which are also established secondary LOPs, including *cis*-2-butenal^4^. Full details of these assignments, which are supported by the acquisition of 1D ^1^H-^1^H COSY and TOCSY spectra, are available in section S1 of the Supplementary Material section.

**Figure 2:**
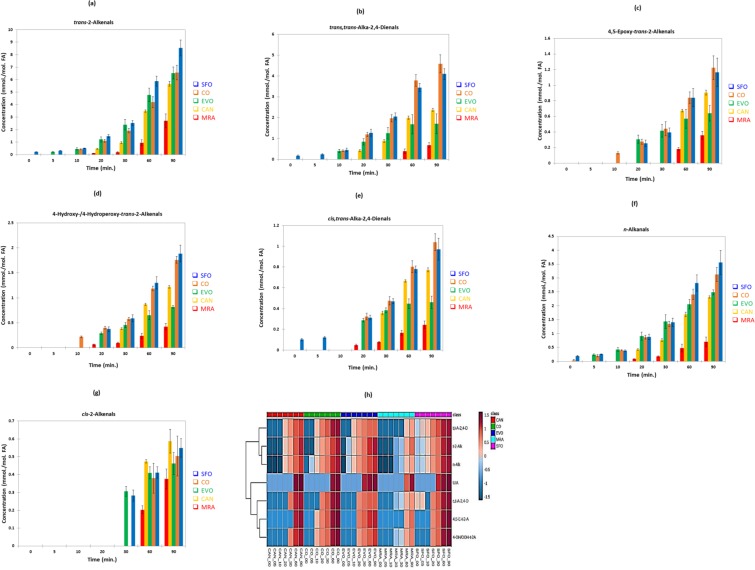
Time-dependence of mean ± SEM concentrations of aldehydic LOPs (mmol./mol. FA) generated in culinary oils investigated when heated according to LSSFEs: (**a**) *trans*-2-alkenals; (**b**) *trans*,*trans*-alka-2,4-dienals, **(c)** 4,5-epoxy-*trans*-2-alkenals; **(d)** combined 4-hydroxy/4-hydroperoxy-*trans*-2-alkenals; (**e**) *cis,trans*-alka-2,4-dienals; **(f)** *n*-alkanals; **(g)** *cis*-2-alkenals. **(h)**, Heatmap diagram showing the time-dependent generation of *trans*-2-alkenals (t-2-Alk), *trans*,*trans*-alka-2,4-dienals (t,t-A-2,4-D), 4,5-epoxy-*trans*-2-alkenals (4,5-E-t-2-A), 4-hydroxy-/4-hydroperoxy-*trans-*2-alkenals (4-OH/OOH-t-2A), *n*-alkanals (n-Alk) and the previously unidentified *cis*-2-alkenal classification (UIA) in sunflower, corn, canola, extra-virgin olive, and MRAFO oils exposed to LSSFEs. Generalised log- (glog-) transformed aldehyde concentrations (mmol./mol. FA) are shown in the right-hand side y-axis: deep blue and red colourations represent extremes of low and high concentrations respectively.

Figure 2 shows plots of mean ± SEM ^1^H NMR-determined total concentrations of each class of aldehyde detected, i.e. (a) *trans*-2-alkenals, (b) *trans*,*trans*-alka-2,4-dienals, (c) 4,5-epoxy-*trans*-2-alkenals, (d) combined 4-hydroxy/4-hydroperoxy-*trans*-2-alkenals, (e) *cis,trans*-alka-2,4-dienals, (f) *n*-alkanals and (g) *cis*-2-alkenals as a function of heating time for sunflower, corn, extra-virgin, canola and MRAFO oils. Corresponding polynomial cubic spline plots of culinary oil aldehyde concentrations *versus* increasing LSSFE time-point are provided in Figures S2(a–g), and these were employed to further explore inter-relationships between the concentrations of each class of these toxins detectable therein and monitored, and also to facilitate the determination of primary and, where appropriate, intermediate lag-phases for their evolution. The thermally-induced generation of *cis-*2-alkenals was found to be very significantly time-delayed, i.e. by 20–30 min. (section S2).

The univariate ANCOVA model applied (equation 2) revealed very highly significant differences between (1) the culinary oils investigated (*p* < 10^−8^, with the exception of *cis*-2-alkenals for which *p* = 4.85 × 10^−3^), and (2) sampling time-points (*p* < 10^−8^) for all aldehydes monitored. Moreover, the oil x sampling time-point interaction effect was also very highly significant for all aldehydes determined, and one of the major critical determinants of this was the significantly higher and lower lag-phase times for the generation of culinary oil MUFA- and PUFA-derived hydroperoxide aldehydic LOP sources respectively; the zero time-point MUFA and PUFA contents of the oils tested varied considerably. However, it should also be noted that the relative evaporative loss magnitudes of the differing aldehydic LOPs generated from either of these sources, i.e. those within >7 aldehydic classes (each containing a range of differing b.pt homologues, as discussed in the Discussion section), is also likely to account for this. Details regarding the *post-hoc* univariate ANCOVA analysis of these datasets are available in supplementary materials section S3.

Figure 2(h) depicts a heatmap diagram showing the time-dependent generation of 7 classes of aldehydes in all oils exposed to the above LSSFEs, and this clearly confirms that the predominantly MUFA-containing MRAFO oil, which contains 91% (w/w) MUFAs and only 4% (w/w) PUFAs (the remainder being SFAs), generates the lowest concentrations of these secondary LOPS, little or none of any of them being formed during the 5–30 min. shallow-frying simulation time-points. Additionally, when compared to PUFA-rich sunflower and corn oils, the lower levels of these peroxidatively-induced aldehydic LOPs produced in the other MUFA-rich oils examined, i.e. extra-virgin and especially canola oils, are also clearly visible. Details regarding agglomerative hierarchal clustering (AHC) and principal component analysis (PCA) of heated culinary oil aldehyde concentrations are provided in section S4, and results arising from these forms of multivariate analyses are displayed in Figure S3 and Table S1 respectively.

It should be noted that in oils with significant or high linoleoylglycerol contents, 4,5-epoxy-*trans*-2-decenal is generated from the further oxidation of *trans,trans-*alka-2,4-decadienal^28^, a specific class of aldehydic LOP arising from fragmentation of the 9-hydroperoxide of linoleate^4,5,7^; therefore, its -CHO function ^1^H NMR resonance appears in heated oil spectra only subsequent to that of its decadienal precursor, i.e. at heating time-points ≥ 20–30 min. (Figures 1, 2 and S2), with the exception of results acquired on corn oil.

Moreover, for the MRAFO product, *trans,trans*-alka-2,4-dienals, 4,5-epoxy-*trans*-2-alkenals, and *cis*-2-alkenals were all undetectable at the 30 min. LSSFE heating time-point (Figs 1, 2 and S2), i.e. these only developed from 60 min. (further details concerning the delayed, time-dependent generation of aldehydes from peroxidised MUFA sources are available in section S3, and estimated primary peroxidative lag phases are provided in Table 1). However, the above primary lag-times were surprisingly long for canola oil, despite its linoleoylglycerol and linolenoylglycerol contents of 17.9 and 10.6 molar % respectively.

**Table 1:** Estimated primary peroxidative lag phases (min.) for the generation of different aldehyde classes in culinary oils exposed to laboratory-simulated shallow frying episodes (LSSFEs) at 180 °C. Primary lag times were estimated by cubic spline analysis, linear interpolation and/or primary quadratic fitting strategies^68^. Peroxidative susceptibility indices (PSIs) of each of the culinary oils tested are also provided. *Indicates that there was some uncertainty associated with this lag time estimate in view of a cyclical response of this aldehyde level to heating time [Fig. S2(e)], and therefore the range provided is somewhat tentative.

Culinary Oil	Peroxidatve Susceptibility Index (PSI)^93^	*Secondary Aldehydic LOP*						
		*trans*-2-Alkenals (δ = 9.48–9.51 ppm)	*trans,trans*-Alka-2,4-dienals (δ = 9.51–9.54 ppm)	4,5-Epoxy-*trans*-2-alkenals (δ = 9.54–9.56 ppm)	4-Hydroxy/4-Hydroperoxy-*trans*-2-Alkenals (δ = 9.56–9.59 ppm)	*cis,trans*-Alka-2,4-dienals (δ = 9.59–9.61 ppm)	*cis*-2-Alkenals (δ = 10.05–10.08 ppm)	*n*-Alkanals (δ = 9.74–9.76 ppm)
Sunflower	62.57	<5	<5	10	10	6–10*	20	6
Corn	63.87	5	5	6	6	11	30	<5
Canola	40.71	12	12	30	20	20	30	10
Extra-Virgin Olive	12.00	<5	5	10	10	10	20	<5
MRAFO	7.40	12	30	30	11	10	30	10

As expected, the MUFA-rich MRAFO, extra-virgin and canola oils generated much lower levels of peroxidised PUFA-derived *trans,trans–*alka-2,4-dienals than those observed in PUFA-rich ones such as sunflower oil (Figs 2 and S2); full details are available in section S2. Moreover, in some cases, the time-dependent production of *trans*-2-alkenals and *n*-alkanals from peroxidised MUFA sources was also found to be preceded by an intermediate lag phase, and for *trans*-2-alkenals, these are particularly notable for the canola and MRAFO oils investigated (20–30 min. in each case). That for extra-virgin olive oil was also visible, but less well defined; plots of total oil *n*-alkanal concentration *versus* heating time also featured this intermediate lag phase. Information detailing the mean concentration orders of magnitude of *trans*-2-alkenals, *trans,trans*-alka-2,4-dienals and *n*-alkanals generated in each oil tested are also provided in section S2.

Table 2 shows the total mean mmol./mol. FA concentrations (of n = 6 replicates) of saturated and α,β-unsaturated aldehydes generated in all oils evaluated at the 20 and 90 min. LSSFE time-points, the former representing an extreme for pan shallow-frying practices. These data again confirm that the MRAFO oil generated the lowest aldehyde concentrations when subjected to simulated laboratory frying episodes. Intermediate levels of total saturated and α,β-unsaturated aldehydes were produced in the other MUFA-rich oils (extra-virgin and canola oils, although each had MUFA contents lower than that of the MRAFO product); however, these were lower than those formed in the PUFA-rich corn and sunflower oils, as expected. At the 20 min. heating time-point, total saturated and α,β-unsaturated aldehyde levels determined in the MRAFO oil were only 9 and 13%, respectively, of those determined in sunflower oil, and only 9 and 14%, respectively, of those in corn oil.

**Table 2:** Total mean ± SEM saturated and α,β-unsaturated aldehyde concentrations (mmol./mol. FA) generated in culinary oils at the 20 and 90 min. LSSFE sampling time-points.

	Aldehyde nature	Saturated (total)		α,β-Unsaturated (total)	
	LSSFE time-point (min.)	20	90	20	90
Culinary Oil	Sunflower	0.88 ± 0.10	3.56 ± 0.42	3.90 ± 0.52	17.22 ± 0.60
	Corn	0.87 ± 0.07	3.13 ± 0.25	3.56 ± 0.26	15.67 ± 0.47
	Extra-Virgin Olive	0.50 ± 0.14	2.48 ± 0.09	3.14 ± 0.29	10.62 ± 0.38
	Canola	0.42 ± 0.02	2.32 ± 0.04	1.80 ± 0.11	11.53 ± 0.14
	MRAFO	0.08 ± 0.01	0.55 ± 0.09	0.51 ± 0.03	4.78 ± 0.80

The time-dependent decline in the PUFA content of each oil was monitored via decreases in the normalised relative intensities of their *bis*-allylic-CH_2_ group resonances (i.e. the δ = 2.73–2.84 ppm ISB, section 4.1). Significant (i.e. ≥5%) decreases in these intensities were observed at 10–20, 10–20, 10–20, 20–30 and 20–30 min. for sunflower, corn, extra-virgin, canola, and MRAFO oils respectively (although the PUFA content of the MRAFO product is very low). Moreover, significant (i.e. ≥5%) reductions in the ^1^H NMR-determined omega-3 FA contents of these oils (section 4.1) were observed at 10–20 min. for corn oil, and 20–30 min. for extra-virgin, canola, and MRAFO oils. For example, our ^1^H NMR data acquired on canola oil indicated that its total omega-3 fatty acid (predominantly linolenate) content was reduced from 10.61 ± 0.03 to 10.60 ± 0.02, 10.57 ± 0.01, 10.53 ± 0.06, 10.19 ± 0.02, 9.25 ± 0.03 and 8.27 ± 0.10 molar % (mean ± SEM) at the 5, 10, 20, 30, 60 and 90 min. LSSFE time-points respectively, i.e. an overall loss of 2.34% (proportionately 22%) of its prior molar content at the extreme 90 min. one.

Consistent with the production of more structurally-complex α,β-unsaturated aldehydes from peroxidised PUFA rather than MUFA sources, ^1^H NMR-determined levels of, for example, 4,5-epoxy-*trans*-2-alkenals, were found to be 1.17 ± 0.24, 1.22 ± 0.15, 0.91 ± 0.06, 0.64 ± 0.13 and 0.36 ± 0.07 mmol./mol. FA (mean ± SEM) at the extreme 90 min. heating time-point in sunflower, corn, canola, extra-virgin olive and MRAFO oils, respectively. Similarly, corresponding mean ± SEM concentrations of *cis,trans*-alka-2,4-dienals monitored at this time-point were 0.97 ± 0.24, 1.04 ± 0.08, 0.77 ± 0.02, 0.46 ± 0.09 and 0.24 ± 0.05 mmol./mol. FA for these oils, respectively.

### ^1^H NMR detection and determination of aldehydic LOPs in samples of fast food restaurant fried potato chip servings (FFRPCSs)

**Figure 3:**
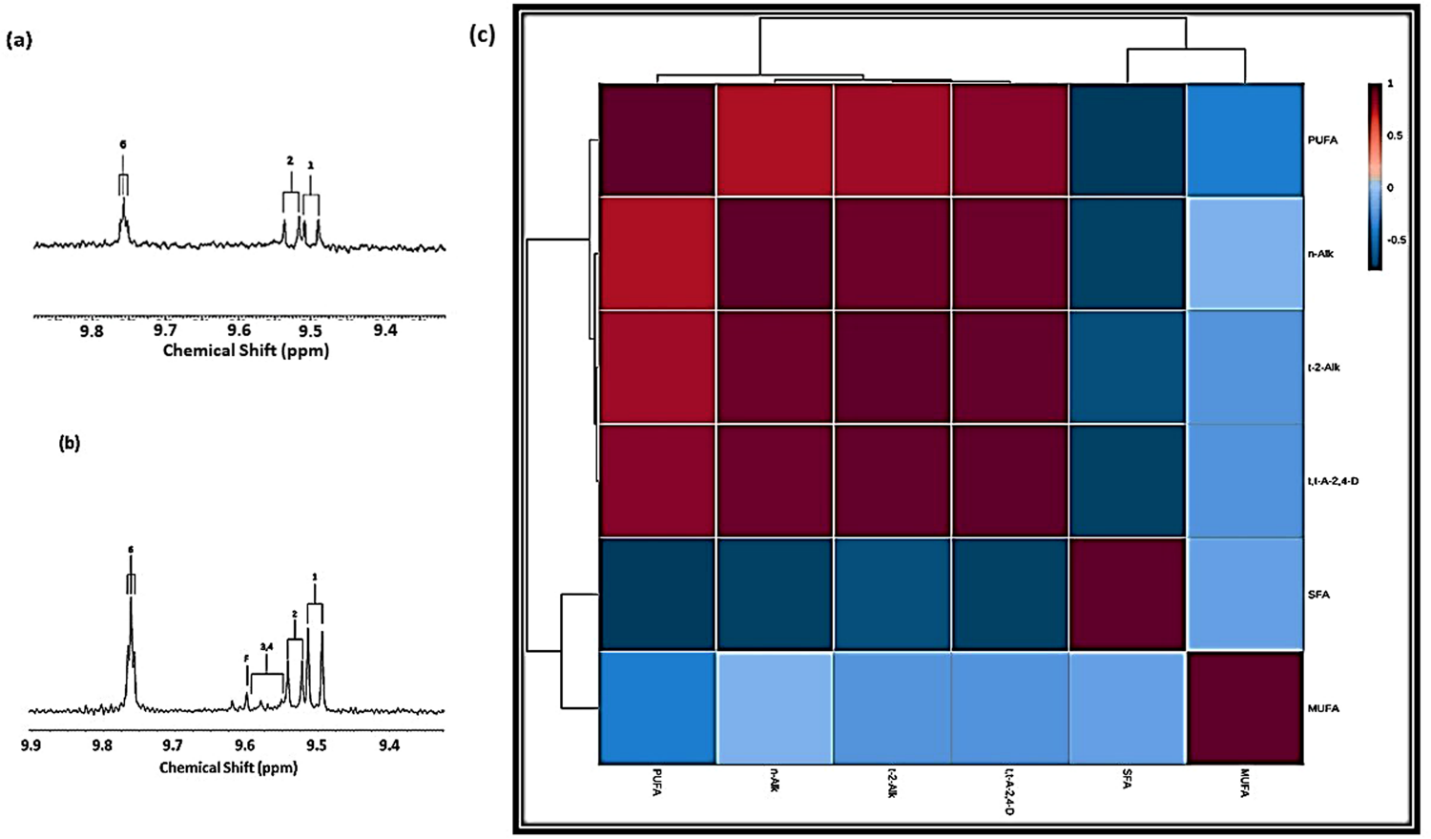
^1^H NMR analysis of aldehydic LOPs in C^2^HCl_3_ extracts of fast food restaurant potato chip samples. **(a)** and **(b)** Expanded aldehydic-CHO proton (9.32–9.90 ppm) regions of the ^1^H NMR spectra of C^2^HCl_3_ extracts of two fried potato chip servings purchased from fast-food restaurants (FFRPCSs), which reveal the presence of *trans*-2-alkenal-, *trans,trans*-alka-2,4-dienal- and *n*-alkanals as major aldehydic LOPs therein. Typical spectra are shown. Abbreviations: as Fig. 1, with F representing formaldehyde in **(b)**. **(c)** Correlation matrix heatmap diagram revealing strong positive correlations between FFRPCS sample *trans*-2-alkenal, *trans,trans*-alka-2,4-dienal and *n*-alkanal concentrations (µmol.kg^−1^, n = 12). Also shown are significant positive correlations between each of these aldehyde classification levels and those of FFRPCS PUFA contents, and negative ones between these and FFRPCS SFA contents, as might be expected. Acylglycerols and aldehydes present in FFRPCS samples predominantly arise from the uptake of heated culinary frying oils by and. Abbreviations: SFA, MUFA and PUFA, saturated, monounsaturated and polyunsaturated fatty acids (as molar % contents); n-Alk, t-2-Alk and t,t-A-2,4-D, *n*-alkanals *trans*-2-alkenals and *trans,trans-*alka-2,4-dienals (as µmol.kg^−1^). Data were generalised logarithmically (glog)-transformed and Pareto-scaled prior to analysis. *Indicates inclusion of all possible PUFAs, including omega-3 and -6 FAs.

^1^H NMR analysis of C^2^HCl_3_ extracts of FFRPCSs confirmed the availability of aldehydic LOPs in these samples, and also provided evidence for their potential passive transfer into these human food sources from the cooking oils in which they had been deep-fried. Typical spectra acquired are shown in Fig. 3, and these contained prominent *trans*-2-alkenal-, *trans,trans*-alka-2,4-dienal- and *n*-alkanal-CHO function resonances, demonstrating the presence of these major classes of aldehydic toxins in FFRPCSs available for human consumption. In some samples collected, relatively minor signals assignable to 4-hydroxy-/4-hydroperoxy-*trans*-2-alkenals and formaldehyde (singlet, δ = 9.60 ppm for the latter) were also visible in spectra acquired, the latter also being a secondary LOP. Table 3 lists the mean ± SEM total concentrations of the three major aldehyde classes (μmol.kg^−1^) found in such FFRPCS samples purchased from 7 different fast-food restaurants (including those from large chain outlets), together with their estimated molar % SFA, MUFA and PUFA compositions, which predominantly reflects those of the oils in which they were fried. The possible identities of these frying media are also listed. Although these mean values for *trans*-2-alkenals and *trans,trans*-alka-2,4-dienals were 30 and 39% of the total aldehyde detectable, respectively, that for *n*-alkanals was 31%. There was also a considerable ‘between-fast-food-restaurant-source’ variation for these FFRPCS determinations (for example, *trans*-2-alkenal contents ranged from 0–345 μmol.kg^−1^).

**Table 3:** Mean ± SEM *trans*-2-alkenal, *trans,trans*-alka-2,4-dienal and *n*-alkanal contents and sample ranges (μmol.kg^−1^) determined in servings of fried potato chips (FFRPCSs) obtained from fast-food restaurants (n = 12), including those available for purchase in large chain outlets. The estimated mean ± SEM molar percentages of total ^1^H NMR-detectable aldehydes found for each class is also provided, as are the molar % fatty acid compositions of these samples, which predominantly reflects that of the oils in which the chip samples were fried. *Indicates inclusion of all possible PUFAs, including omega-3 and -6 FAs. **Indicates the mean of n = 3 or 4 separate replicate C^2^HCl_3_ extractions and determinations. All ^1^H NMR spectra were acquired in duplicate.

Sample Code	Restaurant/Outlet Source Code	Possible Frying Medium Identity	[SFA] (molar %)	[MUFA] (molar %)	[PUFA] (molar %)*	*trans*-2-Alkenals (µmol.kg^−1^)	*trans,trans*-Alka-2,4-dienals (µmol.kg^−1^)	*n*-Alkanals (µmol.kg^−1^)
1	A	Vegetable Oil Blend or High Oleoylglycerol Content Sunflower Oil	11	50	39	50**	51**	84**
2	A	Vegetable Oil Blend or High Oleoylglycerol Content Sunflower Oil	10	52	38	26**	70**	110**
3	B	Lard	47	45	8	trace**	trace**	16**
4	B	Lard	48	43	9	trace**	trace**	12**
5	C	Corn or Soyabean Oil, or Vegetable Oil Blend	18	27	55	87	150	86
6	C	Corn or Soyabean Oil, or Vegetable Oil Blend	20	25	55	175	202	140
7	C	Corn or Soyabean Oil, or Vegetable Oil Blend	20	27	53	142	211	174
8	D	Vegetable Oil Blend or High Oleoylglycerol Content Sunflower Oil	9	50	41	317	529	242
9	E	Lard	53	39	8	33**	30**	75**
10	F	Vegetable Oil Blend or High Oleoylglycerol Content Sunflower Oil	11	54	35	345	295	313
11	G	Corn or Soyabean Oil, or Vegetable Oil Blend	18	28	54	149**	169**	125**
12	G	Corn or Soyabean Oil, or Vegetable Oil Blend	17	28	55	119**	168**	135**
**Mean ± SEM values (range in brackets)**			27.17 ± 5.04 molar % (9–53 molar %)	38.08 ± 3.13 molar % (25–54 molar %)	34.75 ± 6.01 molar % (8–55 molar %)	121 ± 33 μmol.kg^−1^ (range 0–345 μmol.kg^−1^)	157 ± 43 μmol.kg^−1^ (range 0–529 μmol.kg^−1^)	126 ± 25 μmol.kg^−1^ (range 12–313 μmol.kg^−1^)
**Mean % of Total Aldehyde**						30%	39%	31%

However, ^1^H NMR estimates of the total PUFA content of these samples according to the method reported in^9^ also varied substantially (i.e., from 8–55%), as did those of MUFAs (25–54%) and SFAs (9–53%).

Estimated masses of the most predominant *trans*-2-alkenals, *trans,trans*-alka-2,4-dienals and *n*-alkanals produced from the fragmentation of linoleoyl- and linolenoylglycerol CDHPs, and *trans*-2-alkenals and *n*-alkanals from the degradation of oleoylglycerol HPMs, are presented in Table 4 for typical FFRPCS weights of 71, 154 and 400 g. Acrolein mass-adjusted values of these aldehydes are also provided in this Table.

**Table 4:** Estimated aldehyde masses (mg) and contents (ppm) for typical fried potato chip serving sizes of 71, 154 and 400 g (the latter representing an extreme, albeit available one); acrolein mass-equivalent values are provided in brackets. The aldehydes featured correspond to the most predominant ones arising from the thermally-induced, O_2_-mediated peroxidation of oleoyl, linoleoyl and linolenoylglycerol FA sources^29,33,34^.

	Fatty Acid Source	Hydroperoxide Aldehydic Source							
		Oleoylglycerols		Linoleoylglycerols			Linolenoylgycerols		
	Aldehyde	*trans*-2-Decenal	Nonanal	*trans*-2-Octenal	*trans,trans*-Deca-2,4-dienal	*n*-Hexanal	*trans*-2-Pentenal	*trans,trans*-Hepta-2,4-dienal	Propanal
Potato Chip Serving Size (g)	71 g	1.35 (0.61)	1.29 (0.50)	1.09 (0.48)	1.73 (0.64)	0.88 (0.50)	0.75 (0.50)	1.25 (0.63)	trace*
	154 g	2.91 (1.33)	2.80 (1.09)	2.37 (1.04)	3.76 (1.40)	1.91 (1.08)	1.63 (1.08)	2.71 (1.38)	trace*
	400 g	7.57 (2.76)	7.25 (2.87)	6.15 (2.68)	9.76 (3.63)	4.97 (2.80)	4.22 (2.79)	7.05 (3.58)	trace*
Estimated Aldehyde Content (ppm)		18.9 (6.8)	18.1 (7.1)	15.3 (6.8)	24.4 (9.0)	12.5 (7.0)	10.6 (7.2)	17.6 (9.00)	n/a

These estimates are based on the ^1^H NMR analysis of n = 12 FFRPCS samples (Table 3). *Although not ^1^H NMR-detectable in the FFRPCSs explored in these studies (although it, and other short-chain saturated aldehydes, are in selected thermally-stressed oils^9^), traces of residual, non-volatilised propanal are also expected to be found in these servings, particularly those fried in oils containing relatively high levels of linolenoylglycerols, e.g. canola oil. Abbreviations: n/a, not applicable.

There were strong positive correlations between the potato chip contents of each of the 3 major classes of aldehydes detectable. There were also significant positive and negative correlations observed between each of these FFRPCS aldehyde concentrations and the molar % PUFA and SFA contents respectively. There were no significant correlations between each of these aldehyde class levels and molar % MUFA contents. As expected, there was also a strong negative correlation between FFRPCS acylglycerol SFA and PUFA contents. A strong, highly significant correlation between each of these aldehyde class contents and those of total FFRPCS acylglycerols was also found. All these correlations are shown in Fig. 3(c), and a correlation matrix diagram is provided in Table 5.

**Table 5:** Pearson correlation coefficient matrix (with corresponding significance *p* values) between FFRPCS aldehyde levels (µmol.kg^−1^), their total uptaken frying oil acylglycerol contents (mol.kg^−1^), and the molar % polyunsaturated (PUFA), monounsaturated (MUFA) and saturated fatty acid (SFA) constitutions of these potato chip-loaded acylglycerols. Abbreviations: *t*-2-Alk, *trans*-2-alkenals; *t*, *t*-Alka, *trans*, *trans*-alka-2,4-dienals; *n*-Alk, *n*-alkanals; Alka- *n*-Alk AGs, acylglycerols; na, not applicable; ns, not significant.

Aldehyde Class (Concentration)	[*t*-2-Alk] (µmol.kg^−1^)	[*t,t*-Alka-] (µmol.kg^−1^)	[*n*-Alk] (µmol.kg^−1^)	[PUFA] (molar %)	[MUFA] (molar %)	[SFA] (molar %)	Total FFRPCS AGs (mol.kg^−1^)
[*t*-2-Alk] (µmol.kg^−1^)	1.00	0.975 (*p* = 7.45 × 10^−8^)	0.95 (*p* = 3.43 × 10^−6^)	0.79 (*p* = 2.30 × 10^−3^)	−0.27 (ns)	−0.69 (*p* = 1.36 × 10^−2^)	0.97 (*p* < 10^−6^)
[*t,t*-Alka-] (µmol.kg^−1^)	0.975 (*p* = 7.45 × 10^−8^)	1.00	0.96 (*p* = 1.28 × 10^−6^)	0.85 (*p* = 4.06 × 10^−4^)	−0.27 (ns)	−0.76 (*p* = 4.00 × 10^−3^)	0.87 (*p* = 1.69 × 10^−6^)
[*n*-Alk] (µmol.kg^−1^)	0.95 (*p* = 3.43 × 10^−6^)	0.96 (*p* = 1.28 × 10^−6^)	1.00	0.76 (*p* = 4.03 × 10^−3^)	−0.12 (ns)	−0.76 (*p* = 4.00 × 10^−3^)	0.98 (*p* < 10^−6^)
[PUFA] (molar %)	0.79 (*p* = 2.30 × 10^−3^)	0.85 (*p* = 4.06 × 10^−4^)	0.76 (*p* = 4.03 × 10^−3^)	1.00	−0.43 (ns)	−0.80 (*p* = 2.00 × 10^−3^)	na
[MUFA] (molar %)	−0.27 (ns)	−0.27 (ns)	−0.12 (ns)	−0.43 (ns)	1.00	−0.20 (ns)	na
[SFA] (molar %)	−0.69 (*p* = 1.36 × 10^−2^)	−0.76 (*p* = 4.00 × 10^−3^)	−0.76 (*p* = 4.00 × 10^−3^)	−0.80 (*p* = 2.00 × 10^−3^)	−0.20 (ns)	1.00	na
Total FFRPCS AGs (mol.kg^−1^)	0.97 (*p* < 10^−6^)	0.87 (*p* = 1.69 × 10^−6^)	0.98 (*p* < 10^−6^)	na	na	na	1.00

### ^1^H NMR analysis of aldehydic LOPs in potato chips deep-fried in MRAFO, extra virgin olive and sunflower oils in a domestic deep-frying device

**Figure 4:**
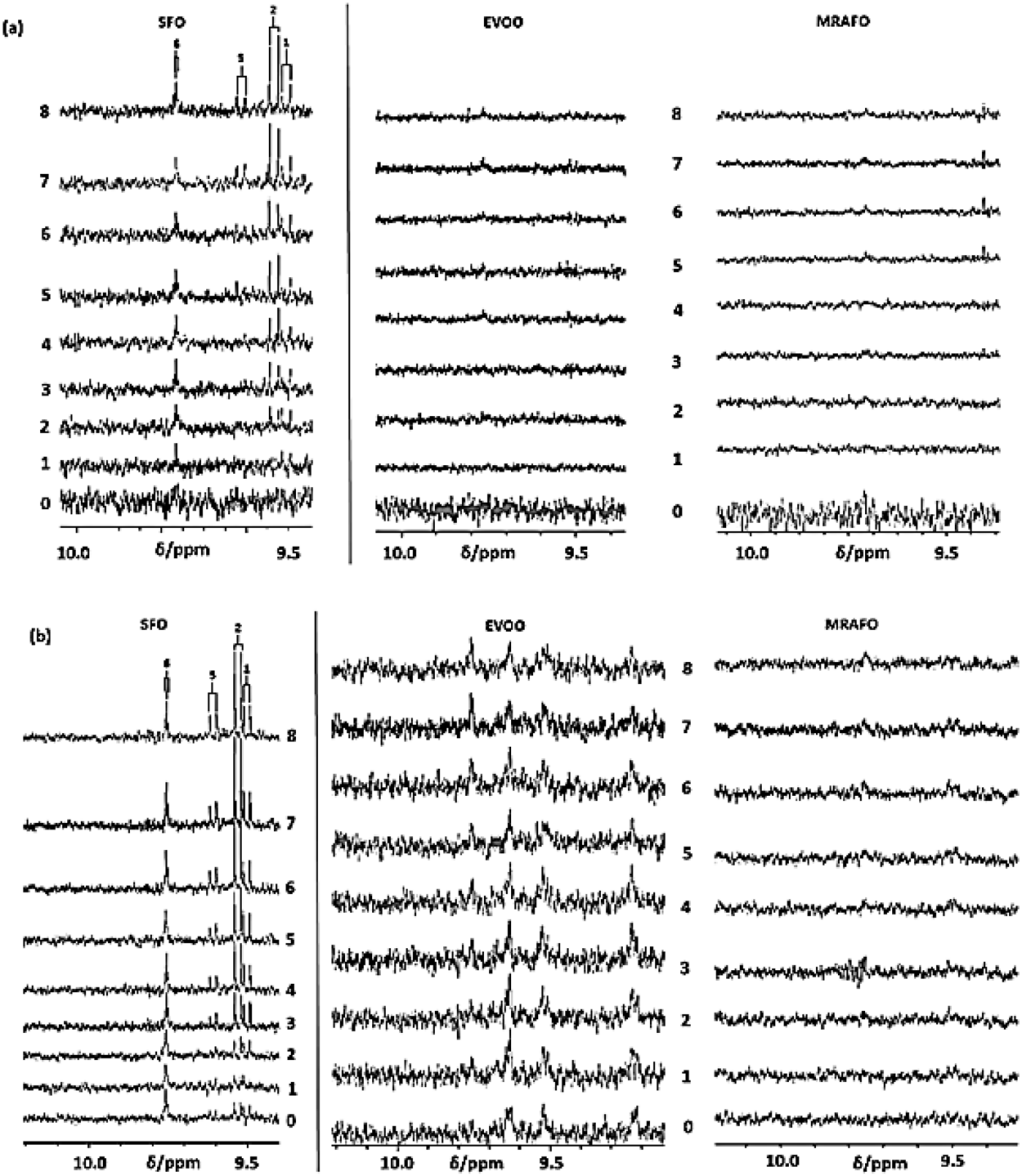
^1^H NMR Analysis of Potato Chips deep-fried according to domestically-based deep repetitive frying episodes (DBDRFEs). **(a)** Partial (9.40–10.10 ppm regions of) ^1^H NMR profiles of C^2^HCl_3_ extracts of potato chip samples deep-fried in sunflower (SFO), extra virgin olive (EVOO) and MRAFO oils exposed to a repetitive cycle of 8 sequential 10 min. duration deep-frying episodes (consecutively labelled 0–8, with 0 representing those acquired on control unfried potato chips) in a commercially-available domestic deep fryer unit at 170 °C. Potato chip samples were fried, collected and extracted by the method described in section 4.5. **(b)** Corresponding partial spectra of the culinary oils employed for these consecutive deep-frying sessions (0–8, as specified above). Abbreviations: SFO, EVOO and MRAFO represent sunflower, extra virgin olive and monounsaturated-rich algae oils respectively; aldehyde-CHO function resonance assignments 1, 2, 5 and 6 correspond to those in Fig. 1.

**Figure 5:**
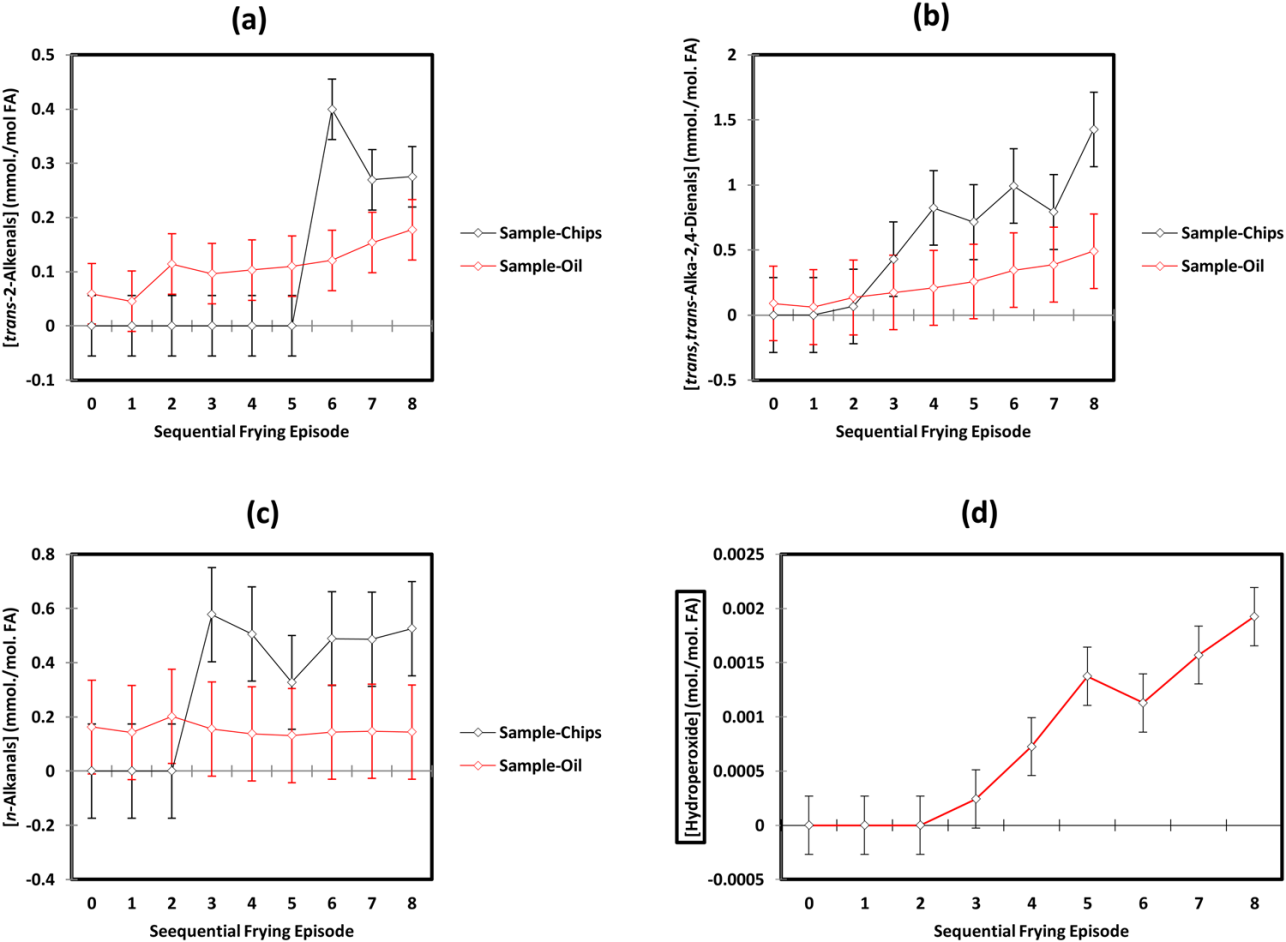
Plots of mean ± 95% CI total FA level-normalised concentrations of **(a)** *trans*-2-alkenals, **(b)** *trans.trans*-alka-2,4-dienals and **(c)** *n*-alkanals present in potato chip (black) and sunflower frying oil (red) samples exposed to consecutive DBRDFEs (0–8) conducted according to the approach described in section 4.5. **(d)**, Corresponding plot of mean ± SEM total lipid hydroperoxide levels (combined CHPDs and HPMs, determined via electronic integration of their broad -OOH proton resonance, δ = 8.13–8.61 ppm) *versus* repetitive frying episode number in sunflower oil. The mean values represent those determined from duplicate potato chip and oil samples collected immediately following each frying episode in the 8 x sequential oil reuse cycle.

Figure 4(a) shows the aldehyde-CHO function regions of the ^1^H NMR profiles of C^2^HCl_3_ extracts of potato chips deep-fried in sunflower, extra virgin olive and MRAFO oils when exposed to a repetitive cycle of 8 consecutive 10 min. deep-frying episodes within a domestic deep fryer device at 170 °C according to section 4.5. Corresponding partial spectra of the oils employed for this purpose are shown in Fig. 4(b). Clearly, increases in aldehyde concentrations detectable in potato chips deep-fried in sunflower oil develop with increasing numbers of frying episodes, and these levels attain maximal (saturation) values for *trans*-2-alkenals, *trans,trans*-alka-2,4-dienals and *n*-alkanals following the 6^th^, 8^th^ and 3^rd^ episodes of the repetitive frying cycle respectively [Fig. 5(a–c)]; their relative percentages were 21, 52 and 27%, 24, 47 and 29%, and 12, 64 and 28% at the 6^th^, 7^th^ and 8^th^ completed consecutive frying session points respectively. These proportions differed somewhat from those observed in potato chip portions obtained from fast-food restaurants (mean value figures were 30, 39 and 31% respectively), although it should be noted that the latter represent only the means of a range of samples fried in differing culinary oil media employed by these outlet sources. Indeed, our deep-fried potato chip level percentages of PUFA only-derived *trans,trans*-alka-2,4-dienals were higher in our sunflower oil-fried chips than the corresponding mean percentage value of the fast-food restaurant ones, as might be expected, especially if such outlets utilised oils of a lower PUFA content than that of sunflower oil, as appeared to be the case for *ca*. 50% of the samples examined (Table 3). Particularly notable is the ^1^H NMR detection of each of these aldehydes in the neat (unheated) oil, with levels of up to 0.15 mmol./mol. FA observed for *n*-alkanals.

However, very little or no aldehydes were detectable in potato chips deep-fried in the extra virgin olive and especially the MRAFO oils under exactly the same conditions [Fig. 4(a)]. Indeed, only barely detectable traces of *n*-alkanals and *trans*-2-alkenals were found in those fried in the latter oil, and only following the 4^th^ deep-frying episode at that.

Similarly, marked increases in CHPD and HPM precursors of aldehydes were observed in sunflower oil with increasing number of repetitive frying episodes [Fig. 5(d)], their generation commencing from the 3^rd^ frying episode and still increasing at the final sampling cycle. Following the 8^th^ frying process, the mean hydroperoxide level was 1.93 mmol./mol. FA, a value equivalent to 6.54 mmol.kg^−1^ oil. However, these hydroperoxides were not ^1^H NMR-detectable detectable in the potato chip samples fried in this medium, and little or none of them were found in the extra virgin olive and MRAFO oil products investigated, nor sequentially collected samples of potato chips fried in these oils.

For consecutive deep-frying episodes with sunflower oil, ANOVA of the total FA content-normalised aldehyde levels performed according to the model provided in equation 3 demonstrated that there was a significant sample (i.e. oil *vs*. potato chip) × sequential deep-frying oil reuse episode number interaction effect for all classes of aldehyde monitored (*p* = 3.82 × 10^−6^, 1.20 × 10^−2^ and 2.07 × 10^−3^ for *trans*-2-alkenals*, trans,trans*-alka-2,4-dienals and *n*-alkanals respectively). These effects are apparent from the plots shown in Fig. 5, in which sunflower oil FA-normalised aldehyde concentrations were either higher than or similar to those found in potato chips during the 0–5th, 0–2nd and 0–2nd repeated deep-frying episodes for *trans*-2-alkenals, *trans,trans*-alka-2,4-dienals and *n*-alkanals respectively, but were greater in the fried potato chip samples than in the oil samples from the 6th-8th, the 3rd-8th and 3rd-8th deep-frying episodes of the cycle for these aldehydes respectively. Statistically significant differences were found between the FA-normalised contents of potato chips and oils at the 6th-8th frying episodes for *trans*-2-alkenals, the 4th, 6th and 8th episodes for *trans,trans*-alka-2,4-dienals, and the 3rd, 4th and 8th ones for *n*-alkanals. However, the oil content of *trans*-2-alkenals was greater than that of potato chip samples on completion of the second episode.

There were statistically significant ‘between-sample nature’ (i.e. oil *vs*. potato chips) differences found for *tran,trans*-alka-2,4-dienals and *n*-alkanals (*p* = 4.42 × 10^−5^ and 5.13 × 10^−3^ respectively), but not for *trans*-2-alkenals. As expected, there were also very highly significant differences observed between consecutive frying episode number for these aldehydes (*p* < 10^−6^, 1.84 × 10^−5^ and 5.13 × 10^−3^ for *trans*-2-alkenals, *trans,trans*-alka-2,4-dienals and *n*-alkanals respectively).

The autocatalytic, sigmoidal dependence of aldehyde concentrations determined in potato chip samples were satisfactorily described by equation 1, where pr1, pr2 and pr3 represent constants (the latter the maximal saturation concentration value), and n = the number of consecutive frying cycle episodes. For these data, R^2^ values demonstrated very good fits of this equation to the experimental datasets (0.96, 0.91 and 0.93 for *trans*-2-alkenals, *trans,trans*-alka-2,4-dienals and *n*-alkanals respectively; pr3 values were 0.27, 1.12 and 0.34 mmol./mol.FA respectively). However, this equation fitted less effectively or poorly to the culinary oil aldehyde concentrations, with R^2^ values ranging from 0.075 for *n*-alkanals to as much as 0.95 for *trans,trans*-alka-2,4-dienals (there appeared to be no significant dependence of oil *n*-alkanal level on frying episode number).

1 $$[{\rm{Aldehyde}}]={\rm{pr}}3/[1+\mathrm{Exp}(\,-\,{\rm{pr}}1-{\rm{pr}}2\ast {\rm{n}})]$$

Pearson correlation coefficients between FA-normalised aldehyde and lipid hydroperoxide concentrations in both potato chip and sunflower oil samples are listed in the matrix provided in Table 6.

**Table 6:** Pearson correlation coefficient matrix (with corresponding significance *p* values) for relationships between FA-normalised potato chip (PC) and frying oil aldehyde levels for the DBDRFE experiments performed. Abbreviations: as Fig. 5, with LOOH representing lipid hydroperoxides.

Sample	Sample	PC	PC	PC	Oil	Oil	Oil	Oil
	LOP (mmol./mol. FA)	[*t*-2-Alk] (mmol./mol. FA)	[*t,t*-Alka-] (mmol./mol. FA)	[*n*-Alk] (mmol./mol. FA)	[*t*-2-Alk] (mmol./mol. FA)	[*t,t*-Alka-] (mmol./mol. FA)	[*n*-Alk] (mmol./mol. FA)	[LOOH] (mmol./mol. FA)
PC	[*t*-2-Alk] (mmol./mol. FA)	1.00	0.61 (*p* = 7.18 × 10^−3^)	0.70 (*p* = 1.22 × 10^−3^)	0.62 (*p* = 6.06 × 10^−3^)	0.80 (*p* = 6.72 × 10^−5^)	0.19 (ns)	0.70 (*p* = 1.13 × 10^−3^)
PC	[*t,t*-Alka-] (mmol./mol. FA)	0.61 (*p* = 7.18 × 10^−3^)	1.00	0.70 (*p* = 1.22 × 10^−3^)	0.59 (*p* = 9.95 × 10^−3^)	0.83 (*p* = 2.03 × 10^−5^)	0.34 (ns)	0.86 (*p* = 5.09 × 10^−6^)
PC	[*n*-Alk] (mmol./mol. FA)	0.70 (*p* = 1.22 × 10^−3^)	0.70 (*p* = 1.22 × 10^−3^)	1.00	0.53 (*p* = 2.37 × 10^−2^)	0.65 (*p* = 3.50 × 10^−3^)	−0.32 (ns)	0.77 (*p* = 1.61 × 10^−4^)
Oil	[*t*-2-Alk] (mmol./mol. FA)	0.62 (*p* = 6.06 × 10^−3^)	0.59 (*p* = 9.95 × 10^−3^)	0.53, (*p* = 2.37 × 10^−2^	1.00	0.715	0.36 (ns)	0.78 (*p* = 1.25 × 10^−4^)
Oil	[*t,t*-Alka-] (mmol./mol. FA)	0.80 (*p* = 6.72 × 10^−5^)	0.83 (*p* = 2.03 × 10^−5^)	0.65, (*p* = 3.50 × 10^−3^	0.72 (p = 8.53 × 10^−4^)	1.00	0.05 (ns)	0.93 (*p* = 1.42 × 10^−8^)
Oil	[*n*-Alk] (mmol./mol. FA)	0.19 (ns)	0.34 (ns)	−0.32 (ns)	0.36 (ns)	0.05 (ns)	1.00	−0.34 (ns)
Oil	[LOOH] (mmol./mol. FA)	0.70 (*p* = 1.13 × 10^−3^)	0.86 (*p* = 5.09 × 10^−6^)	0.77 (*p* = 1.61 × 10^−4^)	0.78 (*p* = 1.25 × 10^−4^)	0.93 (*p* = 1.42 × 10^−8^)	−0.34 (ns)	1.00

The ^1^H NMR profiles of the extra-virgin olive oil samples evaluated demonstrated that they contained low level aldehyde-CHO function signals ascribable to *cis,trans*- and *trans,trans*-alka-2,4-dienals, and also an unassigned resonance located at δ = 9.22 ppm (apparent triplet, conceivably assignable to malondialdehyde, OHC-CH_2_-CHO). However, an *n*-alkanal resonance (*t*, δ = 9.74 ppm) was found to develop with increasing deep-frying episode repetition. No aldehydes were detectable in the MRAFO product prior to its exposure to the 8-fold deep-frying cycle, but very weakly-intense *trans*-2-alkenal and *n*-alkanal signals were observed during the 6^th^–8^th^ sequential episodes [Fig. 4(b)].

### ^1^H NMR investigations of epoxy fatty acid generation in culinary frying oils and fried potato chips

**Figure 6:**
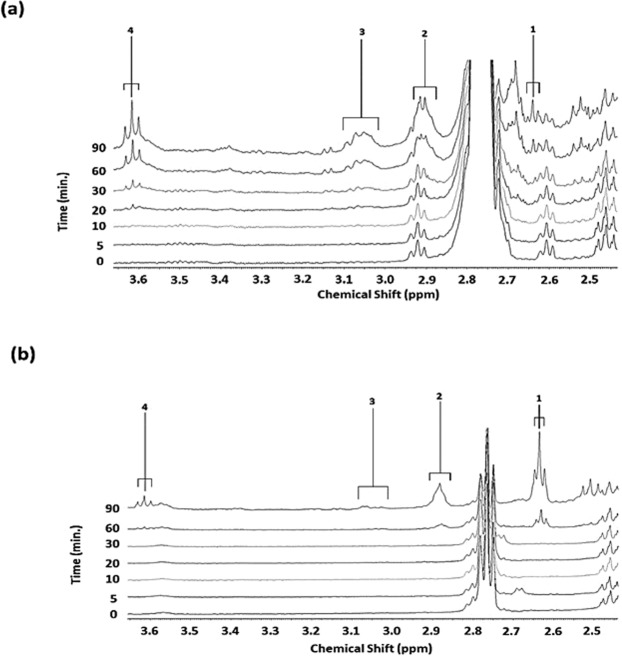
Expanded 2.45–3.65 ppm regions of the 400 MHz ^1^H NMR spectra of **(a)** sunflower and **(b)** MRAFO oils heated according to a LSSFE at 180 °C for periods of 0–90 min. (samples were collected for ^1^H NMR analysis at the 0, 5, 10, 20, 30, 60 and 90 min. time-points). Abbreviations: 1, *trans*-9,10-epoxystearate-CHOHC- protons (δ = 2.63 ppm); 2, a combination of the -CHOHC- protons of 9,10-epoxy-octadecanoate, 9,10-epoxy-12-octadecenoate (leukotoxin), 12,13-epoxy-9-octadecenoate (isoleukotoxin) and *cis*-9,10-epoxystearate (δ = 2.86–2.93 ppm), although almost exclusively the latter LOP in spectrum **(b)**; 3, 9,10-12,13-diepoxyoctadecanoate -CHOHC-CH_2_-CHOHC- functions (δ = 3.07 ppm); and 4, α-CH_2_ group of primary alcohol LOPs (δ = 3.62 ppm).

^1^H NMR resonances assignable to intermediate epoxy acid LOPs were also generated in a time-dependent fashion in the spectral profiles of culinary oils when thermally stressed according to our LSSFEs (Fig. 6 provides examples from the sunflower and MRAFO oil spectra studied). Only *trans*- and *cis*-9,10-epoxystearates were generated in the MRAFO oil, although only at the prolonged, shallow-frying irrelevant 60 and 90 min. time-points. However, as expected, sunflower and the other PUFA-rich oils investigated generated a range of such epoxy acid LOPs, and these were observed from the 30 min. LSSFE time-point (further details are available in section S5).

Also notable was the diminishing intensity of the omega-3 FA (linolenoylglycerol) *bis*-allylic-CH_2_ function resonance in ^1^H NMR spectra acquired on the heated MRAFO oil (*m*, δ = 2.80 ppm), particularly at time points of ≥30 min. As expected, this signal was of only a very low relative intensity in spectra of the unheated MRAFO product in view of its very low content therein [<1% (w/w)].

The above epoxy acid resonances were also observed in ^1^H NMR spectra acquired on potato chip samples purchased from fast-food restaurants (data not shown).

## Discussion

Heating of linoleoylglycerol- and linolenoylglycerol-rich culinary oil products according to LSSFEs generates very high levels of a range of extremely toxic aldehydic LOPs. These established toxins have been proven to be absorbed from the gut into the systemic circulation following their dietary ingestion^10^, where they have the potential to exert a wide range of adverse health effects in humans. In particular, HNE and HHE represent highly toxic and carcinogenic secondary LOPs derived from the thermally-induced degradation of culinary oil PUFAs^3,4,9,29^; indeed, HNE is also a toxic second messenger^30^. However, since these α,β-unsaturated aldehydes predominantly arise from glycerol-bonded linoleate (HNE) and omega-3 FAs (HHE), little or none of them were detectable in thermally-stressed MRAFO and olive oil products (or other PUFA-deplete ones), since these contain only 4 and 5–10% (w/w) linoleoylglycerols respectively, and both products contain ≤1% (w/w) linolenoylglycerols. This also explains why much lower levels of similarly toxic *trans,trans*-alka-2,4-dienals are generated in such MUFA-rich oils when subjected to thermal stressing episodes.

Although significant amounts of aldehydic LOPs also arise from MUFAs, these were only generated at prolonged heating times, i.e. significant lag phases preceded their evolution (Figs 1, 2 and S2, and Table 1). Moreover, only two major classes of aldehydes are produced from the fragmentation of MUFA-derived HPMs (specifically *n*-alkanals and *trans*-2-alkenals, the former of which are arguably of a lower toxicity than the latter), whereas a much broader pattern of these agents are produced from PUFA-derived CHPDs (including *trans,trans*-alka-2,4-dienals, for example)^8,9^. Intriguingly, the total unsaturated aldehyde concentration determined in PUFA-rich sunflower oil heated for a period of 90 min. according to our LSSFEs was very close to or exceeded a staggeringly high figure of 0.05 mol.kg^−1^.

Our results therefore clearly demonstrate that the predominant, >90% (w/w) MUFA source of the MRAFO product renders it much more resistant to thermally-induced, autocatalytic, O_2_-driven peroxidation processes occurring during standard frying practices than frequently employed PUFA-laden ones. Indeed, significantly lower levels of *trans*-2-alkenals and *n*-alkanals were generated in this product at each sampling time-point following exposure to LSSFEs at 180 °C. Furthermore, markedly lower concentrations of PUFA-derived aldehydes, such as *trans*,*trans*-alka-2,4-dienals and 4,5-epoxy-*trans*-2-alkenals, were detected in this product when thermally-stressed in this manner, as expected. Indeed, samples of this oil collected at the 10 and extreme pan-frying 20 min. LSSFE time-points contained little or no toxic aldehydes, whereas substantially higher concentrations of these toxins were found in corresponding samples of PUFA-rich corn and sunflower oils.

Therefore, when used as a medium for shallow frying purposes, this MUFA-rich algae oil serves to offer a high level of protection to human consumers, i.e. it is anticipated that much lower quantities of toxic aldehydes will permeate into food matrices during frying episodes performed with it.

The higher levels of aldehydes found in sunflower, extra virgin olive and MRAFO oils when exposed to our LSSFEs than those found in the ‘at-home’ domestic frying ones (DBDRFEs) are predominantly explicable by the fact that the former processes were performed under shallow frying conditions, whereas the latter domestic ones involved deep-frying, albeit repetitive sessions. Indeed, these results are fully consistent with our previously conducted ^1^H NMR investigations which compared the extent of aldehyde generation within fixed volumes of oils heated according to standard frying practices in vessels of increasing diameter^7^. Concentrations of *trans*-2-alkenals, *trans,trans*-alka-2,4-dienals and *n*-alkanals for the LSSFEs performed were found to be *ca*. 6, 3.5 and 3 mmol./mol. FA respectively for sunflower oil at the 60 min. heating time-point, whereas these values were only *ca*. 0.13, 1.0 and 0.5 mmol./mol. FA respectively following the 6^th^ repetitive frying episode (i.e. 6 × 10 min. sessions with 30 min. cooling periods between each one) performed with this oil according to our domestic deep-frying protocol. Moreover, at the 60 min. heating time-point, although LSSFE levels of *trans*-2-alkenals, *trans,trans*-alka-2,4-dienals and *n*-alkanals were *ca*. 5, 1.7 and 2 mmol./mol. FA respectively for extra-virgin olive oil, and *ca*. 1, 0.3 and 0.5 respectively for the MRAFO product, little or none of these were found in either of these oils when exposed to our DBDRFEs; low concentrations were only detectable in these oils at the later DRDRFE episodes.

Specifically, these results arise from the greater surface area of the frying oil medium during shallow frying practices, and hence a greater exposure of it to atmospheric O_2_ required for the peroxidation process, and also the subsequent dilution of surface-formed aldehydic LOPs into the larger volume frying medium available in the deep-frying experiments conducted.

However, these results are, of course, also explicable by the lower temperature employed for the domestic deep-frying experiments, i.e. 170 rather than 180 °C used for the LSSFEs. A temperature of 170 °C was used for the former experiments since the method of Boskou *et al*.^31^ was followed. A further, albeit less significant, explanation is that chain-breaking antioxidants such as tocopherols in the oil products tested are likely to be more effective at suppressing the peroxidation process under deep- rather than shallow-frying conditions in view of the much lower levels of aldehydic LOPs formed during the former process, i.e. such antioxidants will have an enhanced ability to compete for lipid peroxyl radicals. Additionally, conceivably there will be less volatilisation and thermally-induced degradation of these antioxidants at the lower temperature employed for these domestic deep-frying experiments.

However, the lower levels of aldehydes found in culinary oils exposed to deep-frying processes may, at least in part, be compromised by a greater extent of oil absorption by the fried food available for human consumption.

The marked susceptibility of omega-3 FAs to thermo-oxidation is also very likely to exert a major effect on the omega-6 to omega-3 FA concentration ratio health indices of the cooking oils evaluated here, and this is discussed in more detail in section S6. In view of the very high susceptibility of omega-3 FAs to thermoxidative deterioration, both Belgium and France have sensibly adopted regulations which limit the linolenate content of frying oils to 2% (w/w)^32^.

Intriguingly, the above concentrations of aldehydes arising from the thermal stressing of commercially-available culinary oils is a value representing only that *remaining* therein^3,6–8^. Indeed, a large number of these secondary LOPs generated are volatilised at standard frying temperatures, and this also presents austere health hazards in view of their inhalation by humans, especially those working in fast-food retail outlets or restaurants with insufficient or inadequate ventilation precautions. This is especially the case for secondary LOPs arising from the oxidation of linoleoyl- and linolenoyglycerols. Indeed, a substantial fraction of such aldehydes have boiling-points (b.pts) < 180 °C (at least some of them substantially so), notable examples being *trans*-2-heptenal, *trans,trans*-deca-2,4-dienal and *n*-hexanal (b.pts 165–167, 115 and 131 °C respectively) from peroxidation of the former source^33^, and acrolein, *trans,trans*-2,4-heptadienal and propanal (b.pts 53, 177 and only 49 °C respectively) from peroxidation of the latter acylglycerol. Information regarding the toxicological concerns and actions of inhaled or ingested *trans,trans*-deca-2,4-dienal are provided in section S7.

Contrastingly, major aldehydes derived from the peroxidation of oleoylglycerols include nonanal, decanal, *trans-*2-undecenal and *trans-*2-decenal, which have b.pt values of 194, 213, 234 and 230 °C respectively^33^, and hence this consideration amply, albeit indirectly, serves to reinforce the hypothesis that MUFA-rich cooking oils are much less susceptible to thermally-induced oxidation than PUFA-rich ones, since despite their low b.pts and hence greater volatilities, residual oil *trans*-2-alkenal and *n*-alkanal concentrations in post-heated PUFA-rich oils are always greater or much greater than those observed in corresponding MUFA-rich ones such as MRAFO and, to a lesser extent, canola and olive oils. However, the heating/frying time-dependence of these differences observed is also a critical factor for consideration. Furthermore, an additional aldehyde arising from the fragmentation of HPMs is *n*-octanal, which has a b.pt of 173 °C^33^; hence, presumably somewhat higher concentrations of this LOP would be expected to be found in the gaseous (volatile emission) phase during standard frying practices, whereas higher levels of the above alternative HPM-sourced aldehydes may be anticipated to remain in the thermally-stressed oil medium, together with accessible foods fried therein.

However, Guillen and Uriate (2012)^33^ found that the predominant *trans*-2-alkenals and *n*-alkanals detected in the headspace of extra-virgin olive oil heated at a temperature of 190 °C for 20 hr. in a stainless steel frying tank were *n*-nonanal, and a combination of *trans*-2-decenal and -undecenal, respectively (with significantly higher levels of *trans*-2-decenal than *trans*-2-undecenal being observed). Similar results were found by Fullana *et al*. in 2004^34^, who also observed much lower headspace concentrations of peroxidised linoleoylglycerol-derived *trans*,*trans*-alka-2,4-dienals than those of *trans*-2-alkenals and *n*-alkanals for olive oil when thermally-stressed at 180 °C for a period of 15 hr. in a closed Pyrex Instatherm reaction flask and head.

Here, we have also demonstrated, for the first time, the ^1^H NMR detection of *cis*-2-alkenals in thermally-stressed culinary oils (section S1). Such *cis*- isomers, including *cis*-2-butenal, are also known to be secondary LOPs which arise from the peroxidation of PUFA sources (for example *cis*-2-octenal and -nonenal from linoleate, and *cis*-2-pentenal and -hexenal from linolenate)^2,4^. However, it is conceivable that they may also arise from the thermally-induced isomerisation of their corresponding *trans*-2-alkenals, and this may explain their generation at only the later LSSFE time-points. Indeed, *cis* -2-heptenal may arise from *cis-trans* isomerism of its *trans*-isomer, which is a β-homolysis product of linoleate-12-hydroperoxide^35^. PCA of our oil dataset confirmed a correlation between *cis*- and *trans*-2-alkenal resonances, i.e. they were both found to load significantly and positively on the second orthogonal PC (Table S1). Additionally, 2-heptenal isomers are derived from alka-2,4-decadienal decomposition, along with acetaldehyde, hexanal, acrolein, butenal, 2-heptenal, 2-octenal, benzaldehyde, glyoxal, and *trans*-2-buten-1,4-dial^36^. A further product, *cis*-3-hexenal, may arise via keto-enol tautomerism of a radical combination product of the 1,3-hexadienyl radical arising from the fragmentation of the 12-hydroperoxide of linolenate and hydroxyl radical (^•^OH)^37^. However, we found no major multivariate statistical evidence for associations between the *cis*-2-alkenal ^1^H NMR resonance intensities and those of either *cis,trans*- or *trans,trans*-alka-2,4-dienals. Indeed, this possible precursor may also decompose to 2,3- or 4,5-epoxyaldehydes, which are then further degraded to combinations of either isomeric 2-octenals and acetaldehyde, or glyoxal and 2-octene^37,38^. Although these mechanistic pathways appear to be consistent with Boskou *et al*.’s results^31^, our observations revealed that oil *trans,trans*-alka-2,4-dienal concentrations continued to increase from the 60 to 90 min. time-points for all products explored, with the exception of those for extra virgin olive oil in which it saturated at 60 min.

However, despite strong positive correlations between heated culinary oil *cis*-2-alkenal levels and those of all other aldehydic LOPs (r = 0.86–0.93), the most significant one was that with *trans*-2-alkenals (Fig. S4), and this also indicates that the latter species may isomerise to its *cis*-adduct at 180 °C.

Although the lipid content of fried products is dependent on the types of food, class of frying episode (e.g., shallow- *versus* deep-frying), frying time and frying temperature, these values broadly range from 6–38% (w/w)^39–41^. Moreover, Naseri *et al*.^42^ have reported that the deep frying of fish (silver carp) gave rise to a substantial exchange of acylglycerol (predominantly triacylglycerol) FAs between the food and the culinary oils employed for this purpose, and as expected, the frying oil FA composition substantially altered that of these silver carp fillets on completion of these frying episodes. Comparable results have been observed in similar investigations focused on lipid uptake by potato chips during standard frying practices (e.g.^43^, and reviewed in^40^). For example, the total lipid content of different varieties of fresh, unfried potato tubers is only *ca*. 0.10% (w/w), of which the total PUFA content is 70–76%^44^, but escalates to values exceeding 30% (w/w) in chipped potatoes after frying^40,45^. Hence, frying oil acylglycerol-normalised (proportionate) concentrations of LOPs will also be expected to migrate into foods fried in such media, and in 2012 Csallany *et al*.^46^ found that HNE was readily detectable in French fry samples collected from n = 6 fast-food restaurants at concentrations of 8–32 µg/100 g portion (equivalent to 0.9–4.9 µg/g of extracted lipid).

However, PUFA-derived HNE is always detectable in thermally-stressed PUFA-containing oils at much lower levels than those of similarly health-threatening *tran*s-2-alkenals and *trans,trans*-alka-2,4-dienals - from our laboratory, typical estimates of total fatty acid concentration-normalised total 4-hydroxy-*trans*-2-alkenals/4-hydroperoxy-*trans*-2-alkenals expressed as a molar percentage of the total α,β-unsaturated aldehyde content remaining in oils when heated at 180 °C for a 90 min. period were found to be 10–12% for sunflower, corn and canola oils, and only 7–8% for extra-virgin olive and MRAFO oils (these values represent extreme upper limits for 4-hydroxy-*trans*-2-alkenals in view of the overlap of their -CHO function resonance with that of their 4-hydroperoxy- precursors). These observations are consistent with previous investigations^33^, in which HNE was detectable in sunflower oil at concentrations of *ca*. 350 and 430 μmol.L^−1^ when thermally-stressed at 190 °C for prolonged 17.5 and 20.0 hr. episodes respectively, whereas neither of these LOPs were observed in extra-virgin olive oil at either of these time-points. Similarly, levels of *trans*-4,5-epoxy-*trans*-2-decenal and 4-oxo-*trans*-2-nonenal were found to be much greater in heated, PUFA-rich sunflower oil than those in a correspondingly-heated extra-virgin olive oil product tested, as expected^29^. Further information regarding the levels of HNE and other hydoxyaldehydes detectable in thermally-stressed culinary oils, and their evaporative loss therefrom, is available in section S8, along with that relating to their availability for human consumption in fried food sources.

Notwithstanding, our ^1^H NMR analysis results clearly demonstrate that much greater levels of *trans*-2-alkenals, *trans,trans*-alka-2,4-dienals, and arguably somewhat less toxic *n*-alkanals, are present in FFRPCSs purchased from fast food retail outlets. Indeed, for peroxidised linoleoylglycerols, the predominant compounds featured within the three major aldehydic LOP classes detectable are *trans*-2-octenal, *trans,trans*-deca-2,4-dienal and *n*-hexanal respectively^4,33^, and assuming that these represent 100% of the above 3 classes of aldehydes, our estimated mean μmol.kg^−1^ values would constitute as much as 1.53, 2.44 and 1.25 mg aldehyde/100 g portions of FFRPCSs, equivalent to 1.1, 1.7 and 0.9 mg per small (71 g), and 2.4, 3.8 and 1.9 mg per large (154 g) servings, respectively (Table 4), values much larger than those previously reported for HNE^46^, as might be expected from our ^1^H NMR determinations of the relative quantities of these aldehyde classes detectable in the thermally-stressed oils explored here. These small and large serving portion masses correspond to those provided by a well-known major commercial fast food chain, although it should be noted that ‘large’ FFRPCSs may comprise masses as high as 300–400 g, including those available in the USA or Europe. Similarly, major *trans*-2-alkenal and *n*-alkanal species arising from the fragmentation of oleoylglycerol hydroperoxides are *trans*-2-decenal and nonanal respectively^4^, and again, if these comprised 100% of these two aldehydic LOP classes, then they would be equivalent to 1.90 and 1.89 mg aldehyde/100 g of FFRPCS, corresponding to 1.4 and 1.3 mg per 71 g, and 2.9 and 2.8 mg per 154 g servings, respectively (Table 4). Although propanal has a high volatility, determinations of its concentration as a secondary LOP remaining in used frying oils, and its potential uptake by fried foods could, at least in principle, serve as a marker for the peroxidation of linolenoylglycerols present in frying oils. However, further, more extended studies are required to explore relationships between these fried food LOP contents and the % fatty acid compositions of the corresponding cooking oils employed for deep frying purposes by fast-food retail outlets, or alternative sources of such products.

Also notable is the higher mean molar percentage levels of *n*-alkanals when expressed relative to that of total aldehydes present in these FFRPCSs (*ca*. 30%) than those of the oils in which they are fried (*ca*. ≤ 25%). This may reflect the lower reactivities of *n*-alkanals than α,β-unsaturated aldehydes towards free, and/or protein-incorporated amino acids with selected ‘target’ side-chain amino or thiol functions, processes involving Maillard and/or Michael addition reactions^10,47^ (section S9).

In view of these observations, very recently the Australian Government Department of Health (AGDH) specified that the acceptable daily intake of the similarly-toxic, simplest α,β-unsaturated aldehyde acrolein, i.e. that which is considered to be a level of intake of this molecule that can be ingested daily over an entire lifetime without any appreciable risk to health, to be only 0.5 µg per kg of body weight, i.e. a total of only 35 μg for an assumed (average) human body weight of 70 kg^48^. This alone is a critical toxicological concern, especially since we have found here, for the first time, substantially greater contents of *trans*-2-alkenals, *trans,trans*-alka-2,4-dienals and *n*-alkanals present in FFRPCSs available to consumers for purchase in fast food restaurants. Indeed, assuming that linoleoylglycerol CHPD-derived *trans*-2-octenal represents the total *trans*-2-alkenal content therein, this estimate for a 154 g ‘large’ serving portion of this fried food (*ca*. 2.4 mg) is 68-fold greater than that of this acceptable daily intake limit for its lower homologue acrolein (a value corresponding to 30-fold greater for its acrolein mass-equivalent). Strikingly, this 2.4 mg value is that of only one of the 3 major classes of such cytotoxic/genotoxic aldehydes detectable at similar levels, together with at least several minor ones. Corresponding estimates for the major *trans,trans*-alka-2,4-dienal (*t,t*-DDE) and *n*-alkanal (*n*-hexanal) species arising from linoleoylglycerol peroxidation were > 3.7 and 1.9 mg (acrolein mass-equivalent values of 1.50 and 1.11 mg), respectively, per 154 g serving. Moreover, these estimates correspond to only one potato chip portion of a single fried meal!

The health-threatening significance of these estimated LOP intake values are further exemplified by the World Health Organisation (WHO)’s tolerable intake level of acrolein, which in 2002 was specified as a higher 7.5 μg (0.13 μmol) per day per kg of body weight (equivalent to a burden of only 525 μg per day for an average human body weight of 70 kg)^49^. This tolerable intake level is *ca*. 4.5-fold less than that estimated for only the total *trans*-2-alkenal content of the above 154 g single FFRPCS, although this is only one of the 7 or more classes of aldehydes detected and monitored in this work. Moreover, the estimated mean molar % of this aldehyde was only an estimated 30% of the total aldehyde concentration found in FFRPCSs; that for similarly-toxic *trans,trans*-alka-2,4-dienals was 39 molar % (Table 3). However, although when adjusted for the higher molecular mass of *trans*-2-octenal (126.12) over that of acrolein (56.06), the estimated tolerable daily intake level for an average human would be 1.18 mg, and therefore the content of the former still exceeds this tolerable intake value > 2-fold. Moreover, the ‘acrolein-mass equivalent’ content of total α,β-unsaturated (i.e. both mono- and di-unsaturated) aldehydes would be approximately 3-fold higher than this maximum tolerable intake value.

Our estimated mean level of total *trans,trans*-deca-2,4-dienals present in FFRPCS samples pre-fried in a hypothetical oil containing a 100% (w/w) linoleic acid content (24 ppm) is comparable to that determined in French fries exposed to repeated frying episodes using sunflower oil in a domestic deep-fryer (up to 11 ppm after 3–4 fryings)^31^. As expected, similar levels of this α,β-unsaturated aldehyde were found when this food was fried in PUFA-rich vegetable shortening, but intermediate ones were observed when cottonseed oil was employed in place of sunflower oil, and lower values still were found for palm and olive oils, with olive oil giving rise to the lowest ones (i.e. a maximum level of 3 ppm, consistent with our results). Our higher estimated total *trans,trans*-alka-2,4-dienal level of 157 ± 43 µmol.kg^−1^ (mean ± SEM) is not dissimilar to Boskou *et al*.’s^31^ maximal deep-frying value of *ca*. 65 µmol.kg^−1^ (10–11 ppm) for *trans,trans*-deca-2,4-dienal in French fries. However, our higher estimated mean value will, of course, also include contributions from alternative *trans,trans*-alka-2,4-dienals such as linolenate hydroperoxide-derived *trans,trans*-nona-2,4-dienal, and *trans,trans*-hepta-2,4-dienal (the latter arising from scission of linolenate’s 12-hydroperoxide), in the FFRPCS samples investigated, although the linolenate content of sunflower oil is negligibly small. Moreover, this difference observed may also reflect continued sequential reuse of UFA-rich frying oils in the fast-food outlets from which they were purchased. Our estimated FFRPCS total mean *trans,trans*-alka-2,4-dienal content value is also similar to that found in a further report^50^.

Results acquired from our domestic deep-frying experiments clearly show that only PUFA-laden sunflower oil gave rise to the availability of significant levels of aldehydes for human consumption in repeatedly-fried potato chips, and these levels were similar to those found for 2,4-decadienal by Boskou *et al*.^31^ i.e. 61–88 µg/g of absorbed oil from the 2nd to the 8th frying sessions, equivalent to 401–578 µmol.kg^−1^ oil; assuming an overall 15% (w/w) absorption uptake of sunflower oil frying medium into this fried food^31^ would yield potato chip mass-normalised contents of 60–87 µmol.kg^−1^. Our results also confirmed that MUFA-rich oils such as extra virgin olive and especially MRAFO oils offer little or no toxicological threats to human health when employed for such deep-frying practices.

The oil reuse lag periods observed in Fig. 5(a–c), i.e. those determined from potato chip aldehyde levels only, were 5 episodes for *trans*-2-alkenals, 1-2 for *trans,trans*-alka-2,4-dienals, and 2 for *n*-alkanals respectively, and hence for each of these aldehyde classes, these values provide an indication of the maximum levels of repetitive use for sunflower oil when used for frying purposes according to our domestic deep-frying protocol.

However, it appears that the use of sunflower oil for a maximum of 2 episodes under our deep-frying experimental conditions would not give rise to any significant levels of each aldehyde in fried potato chips, and therefore would not present any adverse toxicological or health concerns. However, our data provide evidence that the additional reuse of this and perhaps other PUFA-rich oils does indeed give rise to toxicologically-relevant concentrations of aldehydes in this commonly consumed fried food. Although significant levels of lipid hydroperoxides were found to be generated in this culinary oil, which sequentially increased with increasing number of repetitive frying episodes performed, they were not detectable in any of the DBRDFE potato chip samples collected, an observation which indicates that they are rapidly degraded to secondary LOPs such as aldehydes and/or epoxy fatty acids, or further products, when uptaken by this food during the frying practice employed. Indeed, such decomposition is likely to be promoted by the availability of catalytic trace levels of transition metal ions [such as those of iron (69–85 ppm) and copper (4–6 ppm)^51^] in potatoes.

Moreover, the significantly higher FA content-normalised aldehyde concentrations observed in the potato chip samples than those of fried oils collected at the later stages of the consecutive deep-frying cycle are conceivably ascribable to the potentiation of UFA peroxidation following their uptake from the frying oil medium by the availability of water and the above levels of catalytic trace level transition metal ions therein. Further investigations are required to explore this phenomenon in detail.

Possible links between the human ingestion of aldehydic LOPs and the development and progression of non-communicable diseases (NCDs) are strongly supported by the establishment of powerful causal links between coronary heart disease (CHD) risk and the more frequent consumption of fried foods, i.e. ≥4 times per week^52^. However, noted limitations of this previously-reported investigation were an insufficient provision of information regarding fried food class stratification, the culinary oils employed by study participants for frying purposes, the frying reuse status of these oils, frying processes (i.e. deep *vs*. shallow pan-frying practices), frying temperature and duration, together with only a limited consideration of overall dietary patterns of the populations investigated. Clearly, all these factors will exert a major influence on (1) the nature and concentrations of LOPs generated in culinary oils exposed to high temperature frying practices, (2) the rate and extent of their passage into fried foods such as potato chips, and their longevity therein, and consequently (3) the level of their dietary consumption by humans. From our data, assuming that all dietary aldehydic LOPs arise from the thermo-oxidation of linoleoylglycerol sources (overwhelmingly the most common culinary vegetable oil PUFA), and also that the most predominant aldehyde homologues in each of the 3 major classes were responsible for the total determined, then consumption of 4 × 154 g fried potato chip servings per week yields estimated mean daily intakes of 1.09, 1.35 and 2.15 mg of *n*-hexanal, *trans*-2-octenal and *trans,trans*-deca-2,4-dienal respectively (0.6, 0.6 and 0.8 mg acrolein mass-equivalents respectively), i.e. 57% of the 154 g potato chip portion values provided in Table 4. Approximately 70% of the above estimated daily aldehyde intake levels comprise the more toxic α,β-unsaturated classes.

The total FA contents of French fries varies widely, and generally ranges from 5 to >15% by weight^39,40^, whereas for standard deep-fried potato chips these values are considerable, i.e. up to 35% (w/w) or more of surface and food microstructure-penetrated acylglycerols^53^. For one major fast food retail chain, the fat content of a 154 g serving of French fries is 25 g [16.2% (w/w)], of which 3 g represents SFAs. Therefore, 4 portions of these fries consumed per week (mean 0.57 portion per day) would constitute an overall fried food fat intake of *ca*. 19% of the reported 77 g mean total daily human UK fat consumption^54^ (14.3 g/day), which would predominantly comprise thermally-peroxidisable UFAs for this particular food service outlet, i.e. *ca*. 16%, corresponding to 12.6 g/day.

Strikingly, Panwar *et al*.^55^ found that CHD patients reported a much elevated, highly statistically significant daily consumption of both deep- and shallow-fried foods (15 ± 25 and 24 ± 60 g respectively) than that of an age-matched healthy control group (1 ± 5 and 3 ± 17 g respectively).

According to Kaliora *et al*.^56^, ingestion of a 150 g serving of French fries which have been deep-dried in sunflower oil and therefore which contain a maximal aldehyde amount of 1.65 mg is sufficient to induce 97% oxidative conversion of LDL *in vitro*.

Previously reported epidemiological, meta-analysis, animal model and laboratory experimental investigations which connect the ingestion of fried foods and/or more specifically, aldehydes themselves (e.g. acetaldehyde and acrolein) to the pathogenesis and/or incidence of further human diseases (including prostate, gastric and breast cancers; autism spectrum disorders and Parkinson’s disease; airways constriction; and hypertensive effects), are outlined in section S10 of the Supplementary Materials section. This section includes information relating to the mechanisms of the toxicities of aldehydes and/or lipid oxidation products (LOPs) present in pre-heated frying oils.

Our results further demonstrate that the shallow- or deep-frying of foods in MUFA-rich, PUFA-deplete cooking oils such as MRAFO, which generate much lower thermally-inducible aldehyde levels in frying oils than those produced in PUFA-rich ones, gives rise to the passage of proportionately much lower concentrations of these toxins into fried food matrices available for human consumption, and therefore such servings offer less potential adverse dietary threats to human health. However, as noted above, a further major factor for consideration is that the b.pts of aldehydes derived from linoleoylglycerol CHPD fragmentation are predominantly lower than those arising from the scission of oleoylglycerol HPMs^33^.

Intriguingly, the above FFRPCS quantities of aldehydes available for human ingestion are not dissimilar to those arising from the smoking of a mean daily allocation of 25 cigarettes, i.e. mg quantities of crotonaldehyde (1.8–5.7 mg), butyraldehyde (2.2–23.2 mg), *n*-hexanal (2.5–9.5 mg) and malondialdehyde (0.24–0.66 mg)^57^. Moreover, the acrolein content of such a cigarette allocation to humans has been estimated to be 0.62–3.5 mg (equivalent to 25–140 μg per cigarette)^58^.

Of course, PUFAs localised within or originating from the food sources themselves will also be expected to undergo thermally-induced oxidative deterioration during frying practices. The conceivable consumption of aldehydic LOPs by amino acids and proteins present in fried foods is outlined in section S11.

An additional consideration is that the concentrations of natural or oil-supplemented lipid-soluble, chain-breaking dietary antioxidants such as α-tocopherol (vitamin E) and DTBHQ, molecules which are known to terminate the autocatalytic lipid peroxidation process, unfortunately appear to be only poorly effective at suppressing the adverse generation of toxic LOPs produced during standard frying practices^7,10^. This is attributable to the poor capacities of such low antioxidant concentrations to combat the aggressive, recycling autocatalytic oxidative assaults upon highly-susceptible PUFAs induced by their exposure to such high temperatures. Along with their chemical consumption by thermally-inducible lipid peroxyl radicals during frying practices, the loss of these antioxidants during such episodes is also ascribable to (1) their volatilisation at such temperatures (tocopherols have b.pts of 200–220 °C, values not much greater than those recommended for standard frying practices), and (2) their thermal instability when exposed to these temperatures (details available in supplementary section S11).

The possible therapeutic intervention of L-cysteine, especially in relation to attenuating acetaldehyde toxicity, in both humans and experimental animals, is available in section S12. Further previously documented interventional and preventative/prophylactic strategies for guarding against oxidative stress induced by the consumption of diets containing peroxidised culinary oils are also documented in this Supplementary Materials section.

A series of ^1^H NMR signals assignable to a range of toxic epoxy acid LOPs were also detectable in samples of culinary oils exposed to LSSFEs. As expected, a multicomponent pattern of these resonances was observed in PUFA-rich sunflower oil when heated according to our LSSFEs at 180 °C for ≥30 min. periods, and these included those assigned to leukotoxin, isoleukotoxin and leukotoxindiol. However, in view of its predominant (>90%) MUFA content, the only ^1^H NMR-detectable epoxy acids found in the MRAFO product evaluated were *trans*- and *cis*-9,10-epoxystearates, although these only evolved at the 60 and 90 min. heating time-points, which are very lengthy and hence irrelevant to those of shallow frying practices (with a *ca*. 20 min. maximal duration). Leukotoxin (9,10-epoxy-12-octadecenoate) and its diol derivative, which can also be generated *in vivo*, are known to give rise to the degeneration and necrosis of leukocytes, and have been implicated in the pathogenesis of multiple organ failure, breast cancer, and perturbations to the reproductive functions of rats^59^. Leukotoxins also exert disruptive effects on cell proliferation and the respiratory burst of neutrophils *in vitro*^60^.

Documented evidence which relates *trans-*fatty acid (TFA) intake to coronary heart diseases (CHDs) remains widespread (albeit somewhat controversial), and their potential health risks in this context are currently considered to be greater than those presented by SFAs^61,62^ (section S13). However, in view of these estimates, it should be stressed that, on a mole-for-mole basis, aldehydes arising from PUFA and MUFA peroxidation are clearly very much more toxic than TFAs, although estimated human intakes of the latter are, of course, much greater than those of the former. However, such investigations focused on the roles of TFAs in promoting CHDs, e.g.^62^, have failed to also consider the myriad of adverse health effects presented by aldehydes ingested in fried food sources, which include atherosclerosis and its pathological sequelae^10,12–14^. Therefore, without any efficient control for such potentially confounding effects, and those also offered by further toxic LOPs (e.g., CHPDs and epoxy acids), along with the quantities of each of these LOP toxins available in human diets, then such public health studies targeting TFAs as CHD ‘malefactor’ molecules may indeed be compromised.

Moreover, in principle TFAs may themselves also be susceptible to peroxidative damage, followed by the possible sequential fragmentation of their corresponding hydroperoxides to toxic secondary LOPs. Despite some major conjecture in the literature available, the heating of oils according to frying practices does not appear to transform natural *cis*-configuration FAs to their corresponding TFA derivatives, although one study has reported a marginal increase in levels of the latter in corn oil following its exposure to stir-frying episodes^63^.

In addition to their greater resistance than PUFAs to thermo-oxidative damage, particularly that induced by high temperature frying practices, dietary MUFAs offer many additional further potential health benefits^64,65^ (further details are available in section S14).

Unless exposed to such frying practices (single or repeated), or alternatively stored and/or exposed to light for prolonged periods of time at ambient temperature, the authors accept that PUFA-rich culinary oils offer little or no threats to human health. Indeed, unperoxidised, intact essential FAs therein such as linoleoyl- and especially α-linolenoylglycerols offer valuable protective health benefits. However, the presence of only trace concentrations of LOP processing contaminants, aldehydic or otherwise, in these products may substantially negate such benefits.

Therefore, a full investigation of all factors exerting an influence on the nature and levels of LOP toxins available in fried foods and hence their roles in the development of NCDs, particularly fried food and CO types used for frying, frying practices (deep or shallow pan frying), temperatures and durations, and oil reuse status, is required. Further considerations should include the extent of fried food consumption prepared at home or at commercial food service outlets, and also the overall dietary patterns of populations surveyed.

## Conclusions

Exposure of PUFA-rich culinary oils to LSSFEs for periods of up to 90 min. generates extremely high levels of hazardous aldehydic LOPs, which may present both serious and chronic threats to human health. Contrastingly, results acquired here also clearly demonstrated that the predominantly MUFA-containing, PUFA-deplete MRAFO oil explored was particularly resistant to LSSFE-induced thermo-oxidation, i.e. much more so than PUFA-rich sunflower and corn oils, and also more so than other MUFA-rich oils tested; the PSI value and [MUFA]:[PUFA] % content ratio of this oil were significantly lower and greater, respectively, than those of the other MUFA-rich oils investigated here. Indeed, little or no toxic aldehydes, nor epoxystearoyl species, were generated in the MRAFO oil at recommended shallow-frying time-points of 5–20 min. Since we have also, for the first time, demonstrated the availability of potentially health-threatening levels of cytotoxic and genotoxic *trans*-2-alkenals, *trans,trans*-alka-2,4-dienals and *n*-alkanals in FFRPCSs (which predominantly arise from passage of thermally-oxidised frying oil media into this food product during deep frying episodes), in principle this MUFA-laden algae oil should present a lower level of health hazards to human consumers than those associated with PUFA-rich oils when employed for this purpose. Indeed, experiments involving the analysis of fried potato chip samples collected during repetitive domestic deep-frying episodes clearly demonstrated that the use of PUFA-rich sunflower oil gave rise to significant reuse-dependent levels of each class of these aldehydes in this regularly consumed food source, whereas only negligible amounts were found in these when MUFA-rich extra virgin olive and MRAFO oils were employed as frying media. Clearly, these results have a high level of public health significance in view of a wealth of evidence available for a myriad of toxicological effects exerted by these secondary LOPs.

## Materials and Methods

### Culinary oil samples

Sunflower, corn, canola, extra-virgin olive and MRAFO oils were all purchased from UK or USA retail stores. Each oil was then de-identified in the laboratory via its transference to coded but unlabelled storage containers. The specified SFA, MUFA and PUFA contents of these oils were 11.0, 28.0 and 61.0% for sunflower oil; 14.4, 23.3 and 61.4% for corn oil; 7.0, 64.4 and 28.5% for canola oil; 13.0, 77.4 and 9.4% for extra-virgin olive oil; and 4.0, 91.2 and 4.2% (w/w) respectively for MRAFO. [MUFA]:[PUFA] % content ratios for these oils were 0.46, 0.38, 2.26, 8.23 and 21.71 for sunflower, corn, canola, extra-virgin olive and the MRAFO products investigated respectively. The molar percentage of omega-3 FAs in these samples was estimated by a previously reported ^1^H NMR method^66^, which involves expression of the intensity (I) of the intelligently-bucketed omega-3 FA chain terminal-CH_3_ function resonance (triplet, δ = 0.97 ppm) chemical shift bucket to that of the total FA chain terminal-CH_3_ signals [i.e. I_0.97_/(I_0.90_ + I_0.97_)], the δ = 0.90 ppm one representing that for all non-omega-3 FAs. In this manner, the molar percentage omega-3 FA (predominantly linolenic acid) contents of these oils was found to be 0.20, 1.89, 10.61, 1.37 and 0.92 molar % for the sunflower, corn, canola, extra-virgin olive and MRAFO oil products tested, respectively. Where appropriate, correction was made for the interfering ^13^C satellite resonance of the major terminal-CH_3_ one, i.e. especially for COs with low or very low omega-3 PUFA FA contents.

The MRAFO oil purchased was supplemented with 1,000 ppm (1.00 g.kg^−1^) of the antioxidant product Fortium^®^ brand MT70 IP liquid, which contained a mixture of α-, β-, γ- and δ-tocopherols [8–14%, 0.5–2%, 43–49% and 11–18% (w/w) respectively] in sunflower oil (the latter serving as a further, but low level source of the ^1^H NMR-detectable PUFAs detectable in this oil).

### Laboratory-simulated shallow frying episodes (LSSFEs) and preparation of oil samples for ^1^H NMR analysis

All oils were heated at 180 °C for periods of up to 90 min. according to a LSSFE, and these experiments were conducted by a ‘blinded’ laboratory researcher. Each 90 min. heating cycle was completed n = 6 replicated sessions for all oils investigated. This shallow frying simulation involved the heating of a 6.00 ml volume of culinary oil in an air-dried 250 ml glass beaker within a thermostated silicon oil bath maintained at a temperature of 180 °C throughout the total heating period. Aliquots (*ca*. 0.25 ml) of oil samples were collected at the 0, 5, 10, 20, 30, 60 and 90 min. heating time-points for ^1^H NMR analysis. Immediately following collection, the lipid-soluble chain-terminating antioxidant 2,5-di-*tert*-butylhydroquinone (DTBHQ) was added to each oil sample (at a final added concentration of 2.00 mmol.kg^−1^) in order to block or retard the further generation of aldehydes and their CHPD and HPM precursors during periods of storage and sample preparation at ambient temperature. Samples were prepared for ^1^H NMR analysis within 2 hr. after collection, and were stored in sealed containers within a light-excluded zone whilst awaiting analysis.

### ^1^H NMR analysis

^1^H NMR measurements on the above samples were conducted on a 400 MHz Bruker Avance spectrometer (Leicester School of Pharmacy facility, De Montfort University, Leicester, UK) operating at a frequency of 399.94 MHz and a probe temperature of 293 K. All spectra were acquired as described in^6–8^. Typically, a 0.20 ml aliquot of each oil sample was diluted to a final volume of 0.60 ml with deuterated chloroform (C^2^HCl_3_) containing 3.67 mmol.L^−1^ tetramethylsilane (TMS) and 15.00 mmol.L^−1^ 1,3,5-trichlorobenzene (1,3,5-TCB): the C^2^HCl_3_ diluent provided a field frequency lock, the TMS acted as an internal chemical shift reference (δ = 0.00 ppm), and 1,3,5-TCB (*s*, δ = 7.20 ppm) served as an internal concentration reference standard. These solutions were then placed in 5-mm diameter NMR tubes. Typical pulsing conditions were: 128 or 256 free induction decays (FIDs) using 65,536 data points and a 4.5 s pulse repetition rate, the latter to allow full spin-lattice (T_1_) relaxation of protons in the samples investigated. Resonances present in each spectrum were routinely assigned by a consideration of chemical shifts, coupling patterns and coupling constants. One- and two-dimensional COSY and TOCSY spectra were acquired to confirm ^1^H NMR assignments as previously described^6–8^.

Preprocessing of these ^1^H NMR spectral profiles for the determination of seven classes of aldehyde, and selected epoxy acids, is described in section S15 of the supplementary material section.

From the FA compositions of these oils, their intrinsic peroxidative susceptibility indices (PSIs) were computed as previously described^67^, i.e. PSI = [0.025(% monoenoic FA)] + [1.00(% dienoic FA)] + [2.00(% trienoic FA)] + [4.00(% tetraenoic FA)] + [6.00(% pentaenoic FA)] + [8.00(% hexaenoic FA)]. However, for all oils investigated here, contributions to the PSI from tetraenoic, pentaenoic and hexaenoic FA sources were negligible.

Details regarding the quality assurance/quality control monitoring of our ^1^H NMR analyses of culinary oil and potato chip determinations of aldehydic LOPs are provided in section S12 of the supplementary materials section. These include evaluations of the accuracies and precision of each of these assay determinations, together with corresponding lower limits of detection (LLOD) and quantification (LLOQ) values for *trans*-2-alkenals, *trans,trans*-alka-2,4-dienals and *n*-alkanals. These parameters were evaluated in both neat C^2^HCl_3_ solutions, and also unheated (control) culinary oils either ‘spiked’ with *trans*-2-octenal and *n*-hexanal, or unspiked. ‘Between-frying cycle’ and repeat determination coefficients of variation (CV) for these LOP assays are also provided.

Calibration curves for typical *trans*-2-alkenals and *n*-alkanals (0–600 µmol.L^−1^ and 0.20–50.00 mmol.L^−1^) were linear, with R^2^ values ≥ 0.993 for neat C^2^HCl_3_ solutions, and ≥0.987 for aldehyde-‘spiked’ C^2^HCl_3_-diluted oil media prepared as described above.

### ^1^H NMR analysis of aldehydes in fried potato chip servings purchased from fast-food restaurants (FFRPCSs)

FFRPCSs were purchased from a total of 12 local fast-food restaurants. To accurately weighed quantities of each sample (1.00–6.30 g), 1.00–8.00 ml volumes of C^2^HCl_3_ were added (predominantly *ca*. 1.0 ml per g), and these mixtures were then mechanically homogenised using an electric pestle rotor (Sigma-Aldrich, UK). Subsequently, the homogenates were then centrifuged at 10,000 × g for a period of 10.0 min. at 4 °C. Exactly 0.60 ml volumes of each clear supernatant were removed and then treated with either 0.06 or 0.12 ml aliquots of a 1.00 × 10^−2^ mol./L stock solution of the 1,3,5-TCB internal standard (final concentration 0.84 or 1.54 × 10^−3^ mol./L respectively) in C^2^HCl_3_, and 0.06 ml of a 10.0 mmol.L^−1^ solution of the chain-breaking antioxidant DTBHQ (final concentration 7.69 × 10^−4^ mol.L^−1^), also in C^2^HCl_3_, the mixtures thoroughly rotamixed, and then transferred to 5-mm diameter NMR tubes for analysis. C^2^HCl_3_ extractions of FFRPCSs were performed either singly, or as 3 or 4 replicates of these samples as indicated in Table 4. All ^1^H NMR spectra of these extracts were acquired in duplicate.

The ^1^H NMR detection of the DTBHQ antioxidant in ^1^H NMR spectra of these FFRPCS sample extracts (*s*, δ = 6.59 ppm in C^2^HCl_3_ solution) confirmed that it had been predominantly retained and not consumed via its chain-terminating antioxidant actions during the sample preparation stages of our experiments.

The SFA, MUFA and PUFA contents of these fried food samples was estimated via integration of ISBs featuring their acylglycerol *bis*-allylic-CH_2_-, -CH_2_-CH = CH- and α-CH_2_-CO_2_- function resonances (δ = 2.73–2.88, 1.96–2.13 and 2.26–2.37 ppm respectively), i.e. an adaption of the method described in^9^.

### Experiments featuring ‘at-home’ deep-frying of potato chips

Eight batches of hand-cut chips of lengths and widths of 87.00 ± 1.15 and 12.70 ± 0.36 mm (mean ± SEM) respectively were consecutively fried 8 times using sunflower, extra virgin olive and the MRAFO oils. For this purpose, we used a modification of the approach utilised by Boscou *et al*.^31^. The deep-frying facility employed was a domestic model (EasyPro, Tefal) equipped with a variable thermostat and an inert cross-lined steel mesh for the purpose of lowering the chips into the oil without contacting the fryer’s inner surface. This deep fryer was filled with 3.00 litres of oil according to the manufacturer’s instructions, and 400 ± 10 g of potato chips then deep fried at a temperature of 170 °C for a period of 10.0 min.

The % (w/w) total PUFA, MUFA and SFA contents of the frying oils employed for these deep-frying studies were 61.0, 28.0 and 11.0%, respectively for sunflower oil, and 30.8, 62.3 and 6.9% respectively for extra-virgin olive oil.

Each frying oil used was allowed to cool for a period of exactly 30 min. between each repetitive frying episode (a total of 8 per full daily cycle). Following each 10 min. frying episode, chips were thoroughly shaken in their wire basket for 15 s, and then allowed to drain therein for 30 s in order to remove excess oil. Subsequently, these chips were transferred to a steel mesh draining board.

For each of the 8 consecutive frying episodes, 2 randomly-selected samples of chips were transferred to plastic-stoppered sample tubes and immediately frozen at a temperature of −20 °C until transported to the laboratory where they were then stored at −80 °C for a maximum duration of 18 hr. prior to ^1^H NMR analysis. Two samples of the unfried (raw) potatoes were also collected and subjected to storage in this manner.

On completion of each frying episode, duplicate samples of each oil were also collected for analysis, and these were also stored prior to analysis in the same manner as the potato chip samples, as were duplicate unheated (control) frying oil samples. Further samples of these oils were collected at a time-point of 10.0 hr. following completion of each repeated frying session.

### Authentic reference aldehydic LOPs

Authentic aldehydes employed for ^1^H NMR reference purposes, including *n*-hexanal, *n*-octanal, *trans*-2-octenal, *trans*-2-nonenal *trans,trans*-deca-2,4-dienal, etc. were obtained from the Sigma-Aldrich Chemical Co. (UK). Crotonaldehyde (butenal) was also purchased from Sigma-Aldrich as a 20:1 molar ratio of its *trans-(E-)*:*cis-(Z-)* isomers in order to permit assignments of resonances for *cis-*2-alkenal LOPs in thermally-stressed culinary oils.

### Experimental design and statistical analysis

For experiments featuring the exposure of culinary oils to LSSFEs, the experimental design for univariate analysis of the total acylglycerol-normalised ^1^H NMR aldehydic class ISB intensity datasets involved an analysis-of covariance (ANCOVA) model, which incorporated 2 prime factors and a total of 3 sources of variation: (1) that ‘between-culinary oils’, qualitative fixed effect (O*i*); (2) ‘between-sampling time-points’ quantitative fixed effect ‘nested’ within ‘culinary oils’ (T*j*); and (3) the culinary oil x time-point first-order interaction effect (OT*ij*). This experimental design is represented by equation 2, in which y_*ijk*_ represents the (univariate) aldehyde ISB dependent variable values observed, μ its overall population mean value in the absence of any significant, influential sources of variation, and e_*ijk*_ the unexplained error (residual) contribution.

2 $${y}_{ijk}={\rm{\mu }}+{{\rm{O}}}_{i}+{{\rm{T}}}_{j}+{{\rm{OT}}}_{ij}+{{\rm{e}}}_{ijk}$$

ANCOVA was conducted with *XLSTAT2016* software. Datasets were generalised logarithmically (glog)-transformed, mean-centred and standardised prior to analysis in order to satisfy assumptions of normality and homoscedasticity. *Post-hoc* analysis of significant differences observed between individual culinary oils and sampling time-points were performed using the Bonferroni method which corrected for false discovery rates.

Agglomerative hierarchal clustering (AHC), principal component analysis (PCA) and Pearson (linear) correlation analysis of the extensive culinary oil NMR-based aldehyde concentration dataset was performed using *Metaboanalyst 3.0* software module options. Datasets (mmol. aldehyde/mol. FA) were analysed either untransformed and unscaled, or alternatively after cube root- or glog-transformations, and Pareto scaling. AHC dendograms were generated employing Euclidean distance and Ward’s linkage clustering algorithm. Heatmap and correlation feature diagrams were also obtained using this software module.

For experiments featuring the domestic deep-frying of potato chips in sunflower oil only, statistical analysis of experimental data was performed according to the ANOVA mathematical model described by equation 3, where y_*ijk*_ represents FA content-normalised sample aldehyde dependent variable values, S_*i*_ and F_*j*_ the ‘between-sample’ (i.e. potato chip *vs* oil levels) and ‘between-sequential frying episode number’ sources of variation (both fixed effects), μ the overall population mean value in the absence of any significant, influential sources of variation, and e_*ijk*_ the unexplained error (residual) contribution.

3 $${y}_{ijk}={{\rm{S}}}_{i}+{{\rm{F}}}_{j}+{{\rm{SF}}}_{ij}+{e}_{ijk}$$

## Supplementary information

SUPPLEMENTARY INFORMATION
