# Supplementary material for: Toxic aldehyde generation in and food uptake from culinary oils during frying practices: peroxidative resistance of a monounsaturate-rich algae oil
Source: Sci Rep. 2019 Mar 11;9:4125. doi: 10.1038/s41598-019-39767-1 (PMC6412032; doi:10.1038/s41598-019-39767-1)
Supplement: Supplementary file 1 — SUPPLEMENTARY INFORMATION [file 41598_2019_39767_MOESM1_ESM.pdf]

Toxic aldehyde generation in and food uptake from culinary oils during frying practices: peroxidative resistance of a monounsaturate-rich algae oil

Sarah Moumtaz, Benita Percival, Devki Parmar, Kerry L. Grootveld, Pim Jansson and Martin Grootveld*

Leicester School of Pharmacy, De Montfort University, The Gateway, Leicester LE1 9BH, United Kingdom.

Supplementary Materials Section

***Author for Correspondence**

Tel:+0044-(0)116-250-6443

Email: mgrootveld@dmu.ac.uk

Section S1. Confirmation of *cis*-2-alkenal aldehydic and vinylic proton NMR assignments using 1D ^{1}H-^{1}H COSY spectroscopy and corresponding *j* values

A clear linkage between the 10.07 and 5.95 ppm signals was also clearly demonstrable in ^{1}H-^{1}H COSY spectra acquired on a range of oil samples thermally-stressed according to our LSSFEs for prolonged, ≥ 30 min. time periods [Figures S1(a) and (b)]. The overall coupling patterns of these vinylic proton resonances were very similar to those of authentic *cis*-2-butenal, although that for the 5.95 ppm oil signal is, of course, expected to have a *ddt* pattern for longer chain *cis*-2-alkenal LOPs. The *j* values for vicinal couplings between these two vinylic ^{1}H nuclei was only 6.9 Hz in ^{1}H spectra acquired on heated oil samples (with a very similar 6.1 Hz value obtained for *cis*-2-butenal in C^2HCl_3 solution), and this confirmed the *cis(Z)*-configuration for this α,β-unsaturated aldehyde; that for the corresponding *trans*-isomer was much greater (*j* = 15.5 Hz), as expected. The minor differences observed between the chemical shift values of authentic *cis*-2-butenal and that present in the ^{1}H NMR profiles of thermally-stressed culinary oils investigated are presumably ascribable to solution matrix effects, i.e. neat C^2HCl_3 for the former, and 1:2 volume ratios of oil:C^2HCl_3 for the latter.

As expected, the ^{1}H NMR spectrum of the butenal (crotonaldehyde) reference sample analysed also contained much more highly intense *trans*-2-butenal resonances, which were consistent with those expected for *trans*-2-alkenals, i.e. δ = 9.48 (*d*) ppm for the -C<u>H</u>O function, and δ = 6.84 (*dq*) and 6.11 (*ddq*) ppm for its 3(β)- and 2(α)- vinylic protons respectively.

Section S2: Mean concentration orders of aldehydic LOP production in culinary oils at the 10 and 20 min. LSSFE sampling time-points: time-dependence of their generation from peroxidised MUFA and PUFA sources

Only selected classes of aldehyde arise from MUFA peroxidation (predominantly *trans*-2-alkenals and *n*-alkanals), and MUFA-rich or -richer cooking oils such as extra virgin and canola oils, in addition to the MRAFO product explored here, gave rise to greater or much greater proportions of these two classes of secondary LOPs when expressed relative to the total aldehyde level generated. Moreover, when generated from MUFA-derived HPMs, these two aldehydic LOP classes are generally only detectable at prolonged LSSFE heating time-points, i.e. ≥ 20 min., as notable for the MRAFO product. Primary lag-times for aldehyde generation, which increase with increasing SFA and MUFA, and decreasing oil PUFA contents, also decrease with the PSI values of the oils tested[93] (Table 1). In general, these lag-phase values were very low for sunflower, corn and extra virgin olive oils (≤ 5-6 min.), intermediate for canola oil (10-12 min.), and high for the predominantly MUFA-containing MRAFO product (20-30 min.) for the most prominent *trans*-2-alkenal, *trans,trans*-alka-2,4-dienal and *n*-alkanal species monitored. Similar results were observed for the 4,5-epoxy- and 4-hydroxy/4-hydroperoxy-*trans*-2-alkenal and *cis,trans*-alka-2,4-dienal signals, although it should be noted that the *cis*-2-alkenal class had a prolonged primary production lag phase for all oils tested, this being 20 min. for sunflower and extra virgin olive oils, and as much as 30 min. for the corn, canola and MRAFO oil products tested.

For the above three most predominant aldehyde classes, the mean concentration magnitude orders of generation at typical shallow-frying sampling time-points of 10 and 20 min. (the latter

commonly employed for chicken strips, for example) were sunflower > corn ≈ extra virgin olive > canola ≈ MRAFO (non-^{1}H NMR detectable in the latter two) and sunflower > extra virgin olive > corn > canola > MRAFO, respectively, for *trans*-2-alkenals; sunflower ≈ corn ≈ extra virgin olive > canola ≈ MRAFO oils (undetectable in the latter two) and sunflower ≈ corn ≈ extra virgin olive > canola > MRAFO oils respectively, for *n*-alkanals. However, for *trans,trans*-alka-2,4-dienals, which only arise from PUFA peroxidation, these orders were sunflower ≈ corn ≈ extra virgin olive > canola > MRAFO oils (undetectable in the latter two), and SFO ≈ corn > extra virgin > canola > MRAFO oils (undetectable in the latter one) for the 10 and 20 min. time-points, respectively. At the 5 min. heating time-point, however, the highest levels of all three of these aldehydes were found in PUFA-rich sunflower oil, and *trans,trans*-alka-2,4-dienals were only ^{1}H NMR-detectable in this product.

Section S3: *Post-hoc* univariate analysis of covariance (ANCOVA) of individual aldehyde class concentrations in culinary oils thermally-stressed according to LSSFEs

Bonferroni-corrected *post-hoc* analysis involving 'between-culinary oil' comparisons of individual aldehyde classes formed during LSSFEs revealed that these mean value differences were in the orders MRAFO < canola < extra virgin ≈ corn ≈ sunflower oils for *trans*-2-alkenals ($p < 10^{-8}$ for all differences observed); MRAFO < canola < extra virgin < corn ≈ sunflower oils for *trans,trans*-alka-2,4-dienals ($p < 10^{-8}$ for all differences observed); MRAFO < canola < extra virgin ≈ corn ≈ sunflower oils for 4,5-epoxy-*trans*-2-alkenals ($p < 10^{-8}$ for all differences observed); MRAFO < canola < extra virgin < corn ≈ sunflower oils for 4-hydroxy/4-hydroperoxy-*trans*-2-alkenals ($p < 10^{-8}$ for all differences observed); MRAFO < canola < extra virgin ≈ corn ≈ sunflower oils for *cis,trans*-alka-2,4-dienals ($p < 10^{-8}$ for all differences observed); MRAFO < canola < extra virgin ≈ corn ≈ sunflower oils for *n*-alkanals ($p < 10^{-8}$ for all differences observed); and MRAFO ≈ canola < extra virgin < sunflower < corn oils for *cis*-2-alkenals (p = 5.00 x 10^{-3}, 2.07 x 10^{-5} and 1.51 x 10^{-4} for the differences observed between MRAFO and extra virgin, corn and sunflower oils, respectively).

Section S4: Multivariate agglomerative hierarchal clustering (AHC) and Principal Component (PC) analyses of the evolution of aldehydic LOPs in culinary oils heated according to LSSFEs

Multivariate agglomerative hierarchal clustering (AHC) analysis of these aldehydic LOP datasets [Figure S4] was performed in order to explore any linkages between the generation of previously unidentified secondary *cis*-2-alkenal LOPs (δ = 10.05-10.08 ppm ISB doublet) and all further aldehydes generated during the thermal-stressing of COs according to our LSSFEs (the former having a delayed development in the heating cycle employed). This AHC analysis provided evidence that the total of 8 classes of aldehydes were optimally clustered into 3 main groups, the first incorporating *trans,trans*-alka-2,4-dienals, *cis,trans*-alka-2,4-dienals, 4,5-epoxy-*trans*-2-alkenals and 4-hydroxy-/4-hydroperoxy-*trans*-2-alkenals (combined ISB for the latter), the second *trans*-2-alkenals and *n*-alkanals, and the third *cis*-2-alkenals alone. Therefore, this analysis did not provide any further information regarding sources or precursors of the secondary *cis*-2-alkenal LOP. However, the clustering of *trans,trans*-alka-2,4-dienals, *cis,trans*-alka-2,4-dienals, 4,5-epoxy-*trans*-2-alkenals and 4-hydroxy-/4-hydroperoxy-*trans*-2-alkenals together is fully consistent with (1) their

common linoleoylglycerol adduct sources, (2) the direct transformation of 5-epoxy-*trans*-2-alkenals from *trans,trans*-alka-2,4-dienals[9], and (3) the possible thermally-induced isomerism of the latter species to *cis,trans*-alka-2,4-dienals. Statistically-significant distinctions between the *cis*-2-alkenals and the other two clusters, and also between the *trans*-2-alkenal/n-alkanal one and that comprising *trans,trans*-alka-2,4-dienals, *cis,trans*-alka-2,4-dienals, 4,5-epoxy-*trans*-2-alkenals and 4-hydroxy-/4-hydroperoxy-*trans*-2-alkenals were found, as indicated in Figure S4. Although ^{1}H NMR analysis at an operating frequency of 400 MHz was unable to distinguish between the -C$\underline{H}$O proton resonances of 4-hydroxy- and 4-hydroperoxy-*trans*-2-alkenals, these aldehydes are expected to be co-clustered since the former secondary LOP acts as a precursor of the latter[9].

Loading scores vectors for a maximum number of two principal components (PCs) isolated from principal component analysis (PCA) of our thermally-stressed culinary oil aldehydic LOP dataset arising from samples exposed to LSSFEs revealed that all aldehydes strongly loaded on PC1 (loadings vectors 0.71-0.89), with the exception of *cis*-2-alkenals, which loaded more strongly on PC2 (loading vector 0.90) than it did on PC1 (Table S1). Although this observation also provides some evidence for the independence of *cis*-2-alkenals from all other ^{1}H NMR-detectable aldehydes, it should also be noted that *trans*-2-alkenals also load strongly on PC2 as well as PC1 (loading vector 0.65 *vs.* a similar value of 0.71 for PC1), and this indicates that *cis*-2-alkenals may arise from the thermally-mediated isomerism of their corresponding *trans*-isomers[29,34].

Section S5: ^{1}H NMR analysis of epoxy acid LOPs in culinary oils exposed to LSSFEs

For sunflower oil, these data revealed the development of multiplet signals assignable to *trans*-9,10-epoxystearate's -C$\underline{H}$O$\underline{H}$C- protons (δ = 2.63 ppm); a combination of the -C$\underline{H}$O$\underline{H}$C- protons of 9,10-epoxy-octadecanoate, 9,10-epoxy-12-octadecenoate (leukotoxin), 12,13-epoxy-9-octadecenoate (isoleukotoxin) and *cis*-9,10-epoxystearate (δ = 2.86-2.93 ppm); and 9,10-12,13-diepoxyoctadecanoate's -CHO$\underline{H}$C-CH_2-C$\underline{H}$OHC- functions (δ = 3.07 ppm) with increasing LSSFE heating time, and these were clearly observable from the 30 min. time-point [Figure 6(a)]. However, unfortunately it was not possible to determine the intensities of these resonances, and hence concentrations, of their corresponding epoxy acids in this oil in view of the marked overlap of those centred at δ = 2.63 and 2.86-2.93 ppm with the relatively highly-intense PUFA *bis*-allylic-C$\underline{H}_2$- function ^{13}C satellite signals. Also notable is a signal located at δ = 3.62 ppm (triplet) attributable to the α-CH_2 group of primary alcohol LOPs; those arising from 9,10-dihydroxy-12-octadecenoate (leukotoxindiol) at δ = 3.43 ppm, and secondary alcohol LOPs at δ = 3.54-3.59 ppm were also discernible in these spectra, but only at the 60 and 90 min. heating time-points.

For the MRAFO oil, however, resonances assignable to both *trans*- and *cis*-9.10-epoxystearates were clearly visible in the ^{1}H NMR spectral profiles acquired, since there was no significant overlap with its very weakly-intense *bis*-allylic-C$\underline{H}_2$ function ^{13}C satellite signal, nor from those arising from leukotoxin or isoleukotoxin, which are generated only from the oxidation of linoleoylglycerol species[9] [Figure 6(b)]. However, these epoxystearates were only visible in spectra of MRAFO exposed to our LSSFEs for periods of ≥ 60 min. ^{1}H NMR-determined mean±SEM concentrations of *trans*- and *cis*-9,10-epoxystearates were 6.21±1.10 and 2.55±0.40

respectively at the 60 min., and 17.50±0.23 and 9.56±1.40 mmol./mol. FA respectively at the 90 min. LSSFE time-point in this oil. The primary alcohol LOP triplet resonance located at δ = 3.62 ppm was also observable in these spectra, but again only at the 60 and 90 min. LSSFE time-points.

Section S6: Potential influence of standard frying practices on the omega-6 to omega-3 fatty acid ratios of culinary oil food sources

A further important consideration is that researchers have previously rationalised and emphasised the critical importance of omega-6 to omega-3 FA ratios in culinary frying oils with special reference to its value as a health index, with 'desirable' values of 2.0-5.1 quoted[S1]. However, α-linolenic acid, the most predominant omega-3 FA in edible plant-derived oils, including those explored here, is *substantially* susceptible to O_2-fueled oxidation during standard frying/cooking episodes (i.e., more than twice that of linoleic acid)), and therefore this ratio is severely limited as a valuable public health index if the FAs ingested by human populations predominantly consist of those present in fried foods laden with thermally-stressed culinary oil lipids, and in which a large proportion of any omega-3 fatty acids present have been peroxidatively degraded during frying practices. Therefore, in view of the relative PSIs of linolenoyl- and linoleoylglycerol species, the omega-3 to omega-6 fatty acid ratios of oils heated according to standard frying practices are expected to be lower, or much lower, than those of corresponding unheated oils. In view of these considerations, it is anticipated that the human diet will contain a significantly or even markedly lower omega-3 FA content than that predicted from the FA composition of unheated oils (along with significantly lower, although presumably less peroxidatively-damaged amounts of linoleic acid). However, the precise frying conditions, time and temperature, along with oil recycling stage, are important factors for consideration, in addition to the FA composition of the cooking oils used and the nature of the food fried therein (which is available for consumption by humans). The relative permeation of differing acylglycerol classes, particularly linoleoyl- and linolenoylglycerols, into fried foods is also an important consideration. For example, our ^{1}H NMR data indicated that the total omega-3 fatty acid content of linolenoylglycerol-rich canola oil was diminished by a mean proportionate value of *ca.* 20 molar % when heated for a period of 90 min. according to our LSSFEs.

Section S7: Toxicological properties of *trans,trans*-deca-2,4-dienal as an inhaled or ingested secondary LOP

Human ingestion of *trans,trans*-deca-2,4-dienal (*t,t*-DDE) derived from linoleoylglycerol peroxidation has been shown to exert cellular toxicity in the liver and kidney[S2], in addition to triggering the proliferation of gastrointestinal epithelial cells[S3]. Since this aldehydic LOP is readily reactive towards DNA base adducts[S4], there is now a major concern that there are highly significant relationships between its ingestion or inhalation and cancer development in humans. Therefore, in view of their ready generation from the peroxidation of culinary oil and food PUFAs, and also their widespread incidence in food products, this class of α,β-unsaturated aldehydes is considered as a major priority by the National Cancer Institute (NCI) and National Toxicology Program (NTP) at the National Institutes of Environmental Health Sciences (NIEHS). Recent epidemiological investigations[S5-S7] have provided strong evidence for an

alarming increase in the incidence of lung cancer in non-smoking women based in China, Hong Kong, Singapore and Taiwan. Moreover, results arising from these studies have demonstrated a powerful association between female lung adenocarcinoma and exposure to cooking oil fumes[S5,S7,S8]. Such fumes are, of course, multicomponent and molecularly complex[S9], and although their compositions vary significantly with frying/cooking oil conditions (including the nature of the frying oil employed and foods exposed to such frying episodes, frying temperature, etc.), aldehydic LOPs are major constituents of this matrix. Indeed, *t,t*-DDE is one of the most predominant peroxidised linoleoylglycerol-derived α,β-unsaturated aldehydes found in these fumes[S10].

Wu and Yen[S11] (2004) have previously reported the induction of oxidative stress and genotoxicty of *t,t*-DDE in human lung carcinoma A549 cells, and in 2005 Chang *et. al.*[S12] reported valuable information regarding its toxicological effects on a non-cancerous human bronchial epithelial cell line (BEAS-2B). The latter study found significant elevations in oxidative stress and ROS generation, together with a significant decrease in glutathione/glutathione disulphide [GSH]/[GSSG] ratio when this cell line was exposed to 1-5 μM *t,t*-DDE short-term (48 hr.), and only 0.10-1.00 μM long-term (up to 30 days). Furthermore, treatment of this cell line with 1.0 μM *t,t*-DDE for a period of 45 days enhanced cell proliferation, and the expression and release of the pro-inflammatory cytokines IL-1B and TNFα. Since there is evidence available that these agents are involved in tumour promotion, these data indicate that *t,t*-DDE, along with other *trans,trans*-alka-2,4-dienals, may indeed promote tumour development in lung epithelial cells.

Section S8: Culinary oil hydroxyaldehyde concentrations and their permeation into fried foods

HNE (4-hydroxy-*trans*-2-nonenal) is only one of several hydroxyalkenal adducts generated during the thermal stressing of culinary oils, further examples including HHE (4-hydroxy-*trans*-2-hexenal) and 4,5-dihydroxy-*trans*-2-alkenals arising from the fragmentation of linolenoylglycerol-derived hydroperoxides. Moreover, HNE has a b.pt of only 89°C[32], and hence its infiltration of fried potato chip food matrices represents that which has not been lost from the oil sample via volatilisation. However, HHE has a b.pt. of 142°C[32], so we might expect that more of it will remain in the culinary oil medium than HNE, and therefore proportionately larger amounts than those anticipated may penetrate into frying foods, although the lower or much lower contents of linolenoylglycerols than those of linoleoylglycerols in commonly-employed frying oils obviously negates this. It should also be noted that 2-hydroxyaldehydes are also lipid peroxidation products arising from linoleate-derived CHPDs, and oleate-derived MHP sources (predominantly 2-hydroxyheptenal and 2-hydroxyalkanals respectively)[S13].

Section S9: Potential depletion of permeated, fried food matrix aldehyde concentrations via reactions with amino acids and proteins: potential bioactivities of Michael addition and Maillard reaction products

Although a significant level of the total aldehydes generated from the oxidation of PUFAs and, to a lesser extent, MUFAs in both thermally-stressed culinary oils and foods fried therein may be depleted by Maillard and Michael addition chemical reactions with proteins and amino acids available in the latter, this still remains a major toxicological concern. Notwithstanding, this

study, together with that conducted by Csallany *et. al.*[46], reports only free levels of aldehydic LOPs available for human consumption, and hence the total amount uptaken by fried potato chip matrices may indeed be significantly, or even substantially, greater. Moreover, these Maillard or Michael addition products may serve as latent sources of bioavailable and bioactive aldehydes following their ingestion. Consistent with this, and on consideration of the facile reversibility of Michael addition reactions of *trans*-2-alkenals with thiols[47] (for example, with the amino acid cysteine available in food sources), β-thiyl-substituted saturated aldehyde adducts arising therefrom potentially represent such latent source of these unsaturated carbonyl compounds, and this process may promote or even prolong their deleterious actions *in vivo* following ingestion.

Section S10: Epidemiological, meta-analysis, animal model and laboratory experimental investigations conecting the ingestion of fried foods and/or aldehydydic LOPs to the pathogenesis and/or incidence of human diseases

Relationships between the consumption of deep-fried foods and prostate cancer risk were explored by Stott-Miller *et. al.*[S14], who found positive associations between ≥ 1 per week intakes of French fries, fried chicken, fried fish and doughnuts and this health risk; many of these estimates were more highly significant for a more aggressive disease status. Moreover, Lippi and Mattiuzza[S15] performed a meta-analysis of published data, and concluded that larger fried food intakes gave rise to an estimated 35% increased risk of prostate cancer.

In 2011, Salaspuro[S16] reported that aldehyde and alcohol dehydrogenase (ALDH2 and ADH respectively) gene polymorphisms are linked to excessive acetaldehyde exposure, and substantially enhance cancer risk in alcohol drinkers; this provides powerful evidence for this saturated aldehyde acting as a local carcinogen in both esophageal and gastric cancers. These observations are clearly relevant to the present study, since acetaldehyde represents a tertiary LOP derived from the degradation of isomeric alka-2,4-dienals[35] or 2,3- or 4,5-epoxyaldehydes[36,37] during high temperature frying processes. This researcher also suggested the L-cysteine, which reacts with and hence neutralises the toxic actions of this aldehyde in the stomach, may provide a novel therapeutic interventional strategy.

Recent key research work performed employed genetically-engineered human cells from patients with a faulty copy of the BRCA2 breast cancer gene in order to explore the mechanisms involved in triggering cancer by aldehyde exposure[S17]. Indeed, this study found that aldehydes give rise to the degradation of cellular BRCA2 protein, and in those with one faulty copy of the corresponding gene (*ca.* 1 in 100 humans), this degenerative effect diminishes this protein's levels below that required for effective DNA repair, a process promoting cancer induction.

Interestingly, Srivastava *et. al.*[S18] examined the genotoxic and carcinogenic risks linked to the consumption of repeatedly-boiled sunflower oil, and discovered that its oral administration to Wistar rats gave rise to a dose-dependent induction of aberrant cells and micronuclei, and significantly depleted antioxidant enzymes. It also altered hepatic foci, along with a significant reduction in hepatic mass.

Woutersen *et al.*[S19] reviewed both animal model and epidemiologic studies focused on the influence of dietary fat consumption on the risks of colorectal, pancreatic, prostate and breast

cancers, and concluded that its effects on prostaglandin and leukotriene biosynthetic routes represented a universal pathway for these adverse health properties.

Further animal studies have focused on the genotoxic and oxidative stress potential of mutagens found in heated soyabean and sunflower oils, and lard[S20], and elevations in liver and blood serum peroxidation parameters, along with changes in lipoprotein compositions were found to be induced in rats by ingestion of thermally-oxidised sunflower oil[S21]. In a further investigation[S22], short-term feeding of pre-heated culinary oils containing strain T100 mutagen and linoleate peroxidation products to rats was found to cause indications of cellular damage to kidneys and liver, and also enhanced urinary mutagenicity and oseophageal cell proliferation.

In 1991, Feron *et. al.*[S23] explored the occurrence, carcinogenic potential, mechanism of action and risk assessment of a series of aldehydes. Although they concluded that acrolein, citral, formaldehyde and vanillin presented no major dietary risk factors, those for acetaldehyde, crotonaldehyde and furfural were potentially significant. They also concluded that such aldehydes should all be subjected to cytogenicity, cytotoxicity and mutagenicity evaluations, with special reference to their extent of human exposure and mechanisms of action.

Xie *et. al.*[S24] recently proposed a mechanistic model of the cytotoxicities of two different broad classes of aldehydes, i.e. that induced by protein and/or DNA damage. Indeed, these researchers proposed a mechanistic model for the cytotoxicities of saturated and α,β-unsaturated aldehydes, i.e. those induced by protein and/or DNA damage, and found that DNA repair processes were essential for diminishing these actions mediated by the former classifications, but not by the latter. Therefore, they concluded that cellular inactivation exerted by the more toxic α,β-unsaturated classes exclusively occurs by protein damage. Moreover, their results indicated that DNA inter-strand crosslinks, but not DNA-protein crosslinks, nor double-strand DNA breaks, represent crucial elements of DNA damage arising from aldehydes. Additionally, this direct DNA damage-independent aldehyde cytotoxicity appears to be driven by the depletion of intracellular, α,β-unsaturated aldehyde-scavenging glutathione (GSH), together with thioredoxin 1 oxidation.

However, LoPachin and Gavin[S25] recently argued that despite aldehydes representing a significant exposural health risk, their mechanisms of toxicity remain poorly understood in view of their structural diversity and differences in chemical reactivity towards critical endogenous biomolecular targets. Indeed, their analyses suggested that both short- and long-chain saturated aldehydes act as ‘hard’ electrophiles, and exert their toxic actions via reactions with the primary amine functions of, for example, protein lysine residue side-chains, whereas α,β-unsaturated classes, further alkenals and α-oxoaldehydes serve as ‘soft’ electrophiles which preferentially react with softer thiolate sulphur-containing functions located on cysteine residues.

Songur *et. al.*[S26] explored the effects exerted by inhaled formaldehyde on the status of oxidants and antioxidants in rat cerebellum during the postnatal development process. This study found that glutathione peroxidase activity, along with malondialdehyde and nitric oxide (NO) levels, were elevated, whereas t-superoxide dismutase activities were significantly diminished in rats exposed to formaldehyde, and that such effects may reflect irreversible oxidative stress and toxicity.

There is now much evidence available that the *in vivo* accumulation of reactive aldehydes serves as a common neurodegeneration mediator in Alzheimer's and Parkinson's diseases, multiple sclerosis and amyotrophic lateral sclerosi[S27,S28]. Indeed, such accumulations in the central nervous system periphery have been ratified for all these neurological disorders.

Moreover, the relationships existing between acetaldehyde and Parkinsonism, with special relevance to the roles of cytochrome P450 and its CYP 2E1 isoenzyme systems, were explored by Vaglini *et al.*[S29]. Indeed, this aldehyde exacerbates the induction of Parkinsonism in mice by the neurotoxin 1-methyl-4-phenyl-1,2,3,6-*tetrahydropyridine*.

Intriguingly, it appears that all hypotheses regarding the pathogenesis of autism spectrum disorder (ASD) are consistent with aldehyde toxicity, featuring the build-up of both endogenous and exogenous forms of these agents in view of mutations in critical aldehyde-consuming enzymes, as with further neurological conditions[S30]. This aldehyde toxicity theory was strongly supported by an intensive review of the biochemical, genomic and nutritional literature, and has a high level of impact regarding the detection, treatment and possibly circumvention of ASD. Indeed, this hypothesis may serve to provide much valuable information regarding distinctions between which neurologically-affected children may be suitable for treatment with micronutrients, and which may not be; to date, genomic investigations have failed to detect dominant genetic errors which are common to all ASD types. Indeed, the toxicity of aldehydes is characterised by cell-localised deficiencies in critical micronutrients, including thiamine (B1), pyridoxine (B6), thiol function-containing antioxidants such as GSH, folate, retinoate, Zn^{2+} and perhaps Mg^{2+} ions, phenomena giving rise to oxidative stress and imbalances in a range of metabolic processes.

Guinea pigs exposed to acrolein and formaldehyde at levels ranging from those occurring in homes and workplaces to excessive ones (0.3 and 0.1-31 ppm respectively) for periods of 2 or 8 hr. demonstrated that a 9 ppm concentration of the latter was required to induce a degree of airway constriction equivalent to that produced by a 2 hr. exposure to 0.30 ppm acrolein[S31]. However, an 8 hr. exposure to only 1 ppm formaldehyde induced an immediate airways constriction similar to that arising from a 2 hr. one to levels of this toxin as high as 9 or 30 ppm. Since aldehyde inhalation gives rise to long-lasting airways effects, these results have a broad public health impact, especially towards humans with sensitive airways, e.g., those with asthma.

Ventaka and Subramanyam[S32] explored the adverse health effects of the consumption of a repeatedly-heated refined vegetable cooking oil on antioxidant enzyme levels, blood biochemistry and histopathological indices of Wistar rats. These studies demonstrated that 3 x consumption of such oils gave rise to significant damage to jejenum, colon and liver of these animals, and also altered the concentrations of selected antioxidant enzymes. Moreover, elevated blood levels of glucose, creatinine and cholesterol, and correspondingly lowered ones of total protein and albumin, were also observed.

Several studies have reported elevated arterial blood pressure indices coupled with further adverse health effects in experimental animal model systems: indeed, Osim *et. al.*[S33] and Leong *et. al.*[S34] found that heated palm oil gave rise to significant blood pressure rises, together with alterations in cardiac muscle in rats. Moreover, Owu *et. al.*[S35] explored effects of the chronic

consumption of diets containing 15% (w/w) fresh or thermally-oxidised palm oil on rat aorta according to standard organ protocols, and concluded that functional changes therein arose from administration of the latter. Moreover, these researchers also found that the dietary consumption of this thermo-oxidatively stressed palm oil diet significantly increased mean arterial pressure (MAP) over those receiving the corresponding unheated oil, an observation associated with elevated LDL and total cholesterol concentrations (which predisposes to high blood pressure). A further study[S36] found that the thermally-oxidised palm oil diet damaged hepatic cells to a greater extent than that observed with the fresh oil product administered.

Section S11: Diminution of α-tocopherol and additional phenolic antioxidants in culinary oils exposed to standard frying practices

In previously reported studies, researchers have found that the α-tocopherol contents of virgin olive and sunflower oils decreased by *ca.* 30 and 15% respectively when heated for a total period of 60 min. at 180°C, with 2 hr. intervals between each of four 15 min. frying cycles[S37]. Furthermore, the total phenolic antioxidant content of the former oil was found to be reduced by *ca.* 50% following exposure to such frying episodes[S38]. In view of the results acquired in this study, the contention that such antioxidants protect against the oxidation of PUFAs present in such oil products is principally only relevant to those that are subjected to prolonged periods of storage at ambient temperature, and not when they are exposed to temperatures associated with frying processes, or indeed more rigorous ones. In this study, although the MRAFO cooking oil tested was supplemented with a mixed tocopherol supplement (1.00 g/L, containing ≥ 70% tocopherols, predominantly γ-tocopherol), these agents are not expected to offer UFAs therein a significant level of protection against thermally-inducible peroxidative damage during the LSSFEs employed, although it would be expected to block such peroxidation during periods of storage, or lower intensity thermal-stressing episodes such as those performed in the DBDRFEs conducted here at 170°C. Similarly, total tocopherol levels in the other oils tested reportedly contain 0.3-1.9 $mmol.kg^{-1}$ (of which 0.2-1.4 $mmol.kg^{-1}$ comprises α-tocopherol)[S39], and again these low levels will not be anticipated to significantly protect PUFAs and MUFAs against peroxidative damage triggered during high temperature frying practices.

Section S12: Interventional and prophylactic strategies to combat the adverse health effects exerted by dietary LOPs in humans and animal model systems: L-cysteine, α-tocopherol and bioflavanoids

With regard to interventional, toxic aldehyde-neutralising prophylactic or therapeutic strategies which may be applied, L-cysteine reacts with carcinogenic acetaldehyde to form chemically-stable 2-methylthiazolidine-4-carboxylate, a process neutralising its toxic actions[S40,S41]. Indeed, oral administration of this semi-essential amino acid was found to successfully reduce salivary acetaldehyde levels during smoking and ethanol consumption regimens[S41,S42]. Moreover, since acetaldehyde is formed from both ethanol and glucose in the achlorhydric stomach, Lindeborg *et. al.*[S43] explored the ability of L-cysteine to eliminate it within the stomach of achlorhydric atrophic gastritis patients following ethanol ingestion, and demonstrated that it significantly decreased its concentration therein. These researchers have therefore suggested that further interventional studies with L-cysteine were required to diminish acetaldehyde exposure in such patients, who have an established high risk of gastric cancer.

In principle, the reactive -CHO functions of both short- and long-chain α,β-unsaturated aldehydes generated in PUFA-rich COs during frying cycles, and ingested as such by humans, may also potentially be removed *in vivo* via reaction with thiols to form thiohemiacetals[S44], although it should also be noted that Michael addition products may also arise from attack of nucleophilic cysteinyl thiolate functions at the electrophilic C3 positions of these aldehyde classes[10,47].

A further interventional strategy of interest is the observation that the teratogenic effects of LOPs present in thermally-stressed safflower oil are at least partially suppressed via the co-administration of α-tocopherol (vitamin E)[20].

An additional circumventing, albeit prophylactic approach available to protect against the deleterious effects arising from the ingestion of dietary LOPs is nutritional supplementation with appropriate antioxidants, and Regulwska-Ilow *et. al.*[S45] investigated the protective effects of bioflavonoids against risks presented by diets containing oxidised fats/lipids by an evaluation of the hepatoprotective activity of a bioflavonoid extract in male Buffalo rats throughout a 4 week period. These researchers found significant elevations in erythrocyte superoxide dismutase activity in groups receiving oxidised lipid diets, amongst other effects, and concluded that the extract tested positively influenced antioxidant systems in rats receiving diets rich in such LOP sources.

A further study[S46] found that the atherogenic indices of laboratory rats were significantly higher for groups consuming unheated lard than those fed with fresh sunflower oil, but higher for those receiving thermally-oxidized oil than those fed oxidized lard, as should be expected from the much higher levels of LOPs present in this oil. However, lard and sunflower oil were both excessively thermally-stressed at 200°C for a period of 35 hr. in this investigation. Dietary supplementation with *Scutellaria baicalensis* bioflavonoids as an interventional strategy successfully decreased the atherogenic index value of the group fed with fresh sunflower oil, although surprisingly increased that of the group fed with oxidized lard.

Section S13: Toxicities and adverse health effects presented by dietary *trans*-fatty acids (TFAs)

Previous conservative estimates by Ascherio *et. al.*[91] have indicated that the replacement of a partially hydrogenated fat in the U.S. diet with natural, unhydrogenated vegetable oils would prevent *ca.* 30,000 premature deaths from coronary heart diseases, although epidemiological evidence from 1994 provides estimates closer to 100,000[S47]. Moreover, in 2009, a meta-analysis of prospective investigations has indicated a 20-33% greater risk of myocardial infarction or coronary heart disease (CHD) death for every 2% TFA consumption energy isocalorifically substituting either carbohydrate, SFAs, and natural *cis*-configuration MUFAs and PUFAs[60]. Evidence for the more powerful CHD-promoting effects of *trans*-18:2 and *trans*-18:1 FAs over that of the corresponding *trans*-16:1 isomer was also presented in this report.

Section S14: Potential health benefits offered by MUFA-rich diets and culinary oils

The potential health benefits offered by MUFA-rich diets are manifold, and a recent systematic review has provided evidence that human blood plasma HDL cholesterol and total triacylglycerol concentrations are elevated and diminished, respectively, subsequent to participants receiving a MUFA-rich diet[62]. Moreover, both systolic and diastolic blood pressure indices have been found to be significantly reduced during short- and long-term dietary regimens involving high MUFA contents. MUFA-rich diets were also found to alleviate glycosylated haemoglobin concentrations, and also to induce hypoglycaemic effects in type 2 diabetic patients[62]. Additionally, although not unanimous, many meta-analysis studies conducted have indicated a reduced incidence of coronary heart disease cases in participants receiving a high MUFA content dietary regimen[62,63].

Section S15: Preprocessing of ^{1}H NMR spectral profiles: Determinations of classes of aldehydic and epoxy acid LOPs in culinary oils, and their lower limits of detection and quantification (LLOD and LLOQ respectively)

Clearly visible aldehydic LOP regions of the spectral profiles acquired (i.e. those within the 5.40-10.20 ppm spectral range) were preprocessed by the application of a separate macro for the 'intelligent bucketing' processing sub-routine. These procedures were conducted using the ACD/Labs Spectrus Processor 2012 software package (ACD/Labs, Toronto, Ontario, Canada M5C 1T4), and this generated a culinary oil dataset matrix consisting of bucket variables (intelligently-selected buckets, abbreviated as ISBs) corresponding to the-C$\underline{H}$O function resonances of a range of aldehyde classes, specifically *trans*-2-alkenals (doublet, δ = 9.48-9.51 ppm), *trans,trans*- and *cis,trans*-alka-2,4-dienals (both doublets, δ = 9.51-9.54 and 9.59-9.61 ppm respectively), 4,5-epoxy-*trans*-2-alkenals (doublet, δ = 9.54-9.56 ppm), 4-hydroxy-/4-hydroperoxy-*trans*-2-alkenals (both doublets, δ = 9.56-9.59 ppm), *n*-alkanals (triplet, δ = 9.74-9.76 ppm), and a further unsaturated aldehyde species (δ = 10.05-10.08 ppm) assigned to *cis*-2-alkenals for the first time in this work. Prior to commencing this intelligent bucketing process, all spectra were examined visually for any inherent distortions and manually corrected, if required. The electronic intensities of resonances corresponding to each of the above -C$\underline{H}$O resonance ISBs were normalised to that of one encompassing all acylglycerol-C$\underline{H}_3$ function signals (δ = 0.82-0.99 ppm) so that their concentrations in each oil sample could be expressed as µmol. or mmol. aldehyde per mol. of FA (mmol./mol. FA). 'Between-frying cycle' sample coefficients of variation for all aldehyde class determinations made on the n = 6 replicated thermal stressing episodes ranged from 3.1-11.7% for all oils investigated, whereas those for repeat determinations made on the same oil sample were ≤ 1.9%.

Similarly, resonances ascribable to the -C$\underline{H}$O$\underline{H}$C- functions of *trans*- and *cis*-9,10-epoxystearates (multiplets, δ = 2.63 and 2.88 ppm respectively); a combination of resonances ascribable to the -C$\underline{H}$O$\underline{H}$C- protons of 9,10-epoxy-octadecanoate, 9,10-epoxy-12-octadecenoate (leukotoxin), 12,13-epoxy-9-octadecenoate (isoleukotoxin) and the -C$\underline{H}$OHC−CHO$\underline{H}$C- protons of 9,10–12,13-diepoxyoctadecanoate (mutliplets, δ = 2.90 ppm); and the -CHO$\underline{H}$C-CH$_2$-C$\underline{H}$OHC- protons of 9,10-12,13-diepoxyoctadecanoate (multiplet, δ = 3.07 ppm)[32] were also preprocessed and their intensities determined in this manner.

Lower limits of detection and quantification (LLOD and LLOQ respectively) values for the typical *trans*-2-alkenal and *n*-alkanal LOPs (*trans*-2-octenal and *n*-hexanal respectively) were determined via the performance of sertial dilutions of these analytes, and a consideration of the 3σ and 10σ signal-to-noise approaches using the *MNova* signal-to-noise ratio (SNR) software module. In neat C^2HCl_3 solution, which is appropriate for the analysis these agents in C^2HCl_3 potato chip extract solutions, LLOD and LLOQ estimates for *trans*-2-octenal were 12 and 38 $\mu mol.L^{-1}$, whereas these values were 10 and 33 $\mu mol.L^{-1}$ for *n*-hexanal. Notwithstanding, as expected, both these parameters significantly improved with the extraction of larger quantities of potato chip samples into smaller C^2HCl_3 volumes as described in sections 4.4 and 4.5. They also markedly improved with the acquisition of 1,024 or 2,048 ^1H NMR scans for samples analysed.

For the analysis of these aldehydic LOPs in oil samples, however, these LLOD and LLOQ values were higher, specifically 51 and 172 $\mu mol.kg^{-1}$ respectively for *trans*-2-alkenals (equivalent to 15 and 51 μmol./mol. FA respectively); 46 and 153 $\mu mol.kg^{-1}$ for *trans,trans*-alka-2,4-dienals (equivalent to 14 and 45 μmol./mol. FA respectively); and 20 and 69 $\mu mol.kg^{-1}$ for *n*-alkanals (equivalent to 6 and 20 μmol./mol. FA respectively) for the 1/3 (v/v) diluted oil samples prepared as outlined in section 4.3. These higher values are ascribable to the influence of significant oil medium matrix effects at 1/3 oil/C^2HCl_3 dilution ratios. As expected, these values improved approximately 2-fold with lower C^2HCl_3 dilution levels of such oils (i.e. 2/3 rather than 1/3 primary dilutions), and also the acquisition of larger numbers of ^1H NMR scans (1,024 or 2,048).

Supplementary Materials Section References

S1. Simopoulos, A. P. The importance of the ratio of omega-6/omega-3 essential fatty acids. *Biomed Pharmacother.* **56**(8), 365-79 (2002).

S2. Hageman, G., Verhagen, H., Schutte, B., & Kleinjans, J. Biological effects of short-term feeding to rats of repeatedly used deep-frying fats in relation to fat mutagen content. *Food Chem. Toxicol.* **29**, 689–698 (1991).

S3. National Toxicology Program, P. H. S., National Institutes of Health, U.S. Department of Health and Human Services (1993). 2,4-Decadienal CAS No. 25152–84–5. Testing Status of Agents at NTP.

S4. Carvalho, V. M. *et al.* 1,N6-etheno-2'-deoxyadenosine adducts from trans,trans-2,4-decadienal and trans-2-octenal. Adv. Exp. Med. Biol. 500, 229–232 (2001).

S5. Ko, Y. C. *et al.* Chinese food cooking and lung cancer in women non-smokers. *Am. J. Epidemiol.* **151**, 140–147 (2000).

S6. Tan, Y. K. *et al.* Survival among Chinese women with lung cancer in Singapore: A comparison by stage, histology and smoking status. Lung Cancer **40**, 237–246 (2003).

S7. Zhong, L., Goldberg, M. S., Parent, M. E. & Hanley, J. A. Risk of developing lung cancer in relation to exposure to fumes from Chinese-style cooking. *Scand. J. Work Environ. Health* **25**, 309–316 (1999).

S8. Zhong, L., Goldberg, M. S., Gao, Y. T. 7 Jin, F. (1999a). Lung cancer and indoor air pollution arising from Chinese-style cooking among non-smoking women living in Shanghai, China. *Epidemiology* **10**, 488–494 (1999).

S9. Shields, P. G. *et al.* Mutagens from heated Chinese and U.S. cooking **o**ils**.** *JNCI J Natl Cancer Inst* **87**(11), 836-841.doi: 10.1093/jnci/87.11.836 (1995).

S10. Wu, S. C., Yen, G. C. & Sheu, F. Mutagenicity and identification of mutagenic compounds of fumes obtained from heating peanut oil. *J. Food Prot.* **64**, 240–245 (2001).

S11. Wu, S. C.& Yen, G. C. Effects of cooking oil fumes on the genotoxicity and oxidative stress in human lung carcinoma (A-549) cells. *Toxicol. In Vitro* **18**, 571–580 (2004).

S12. Chang, L. W., Lo, W-S. & Lin, P. Trans,trans-2,4-decadienal, a product found in cooking oil fumes, induces cell proliferation and cytokine production due to reactive oxygen species in human bronchial epithelial cells. *Toxicol. Sci.* **87**(2), 337-343. doi:10.1093/toxsci/kfi258 (2005).

S13. Loidl-Stahlhofen, A. & Spiteller, G. alpha-Hydroxyaldehydes, products of lipid peroxidation. *Biochim Biophys Acta.* **1211**(2), 156-160 (1994).

S14. Stott-Miller, M., Neuhouser, M. L. & Stanford, J. L. Consumption of deep-fried foods and risk of prostate cancer. *Prostate* **73**, 960-969 (2013).

S15. Lippi, G. & Mattiuzzi C. Fried food and prostate cancer risk: systematic review and meta-analysis. *International Journal of Food Science and Nutrition* **66**(5), (2015).

S16. Salaspuro, M. Acetaldehyde and gastric cancer. *J Digest Dis.* **12**, 51-59 (2011).

S17. Tan, S. L. W., *et. al.* A class of environmental and endogenous toxins induces BRCA2 haploinsufficiency and genome instability. Cell (2017). DOI: 10.1016/j. cell. 2017. 05.010

S18. S. Srivastava, M., Singh, J., George, K. & Bhui, Y. Shukla Genotoxic and carcinogenic risks associated with the consumption of repeatedly boiled sunflower oil. *J Agric Food Chem*, **58**, 11179-86 (2010).

S19. Woutersen, R. A., *et al.* Dietary fat and carcinogenesis. *Mutat Res.* **443**, 111-27 (1999).

S20. Dung, C. H., Wu, S. C. & Yen, G. C. Genotoxicity and oxidative stress of the mutagenic compounds formed in fumes of heated soybean oil, sunflower oil and lard. *Toxicol In Vitro* **20**, 439-47 (2006).

S21. Garrido-Polonio, C., *et al.* Thermally oxidized sunflower-seed oil increases liver and serum peroxidation and modifies lipoprotein composition in rats. *Br J Nutr.* **92**, (2004).

S22. Hageman, G., Verhagen, H. & Schutte, B. Biological effects of short-term feeding to rats of repeatedly used deep-frying fats in relation to fat mutagen content. *J Food Chem Toxicol.* **29**, 689-698 (1991).

S23. Feron, V. J., Til, M. P., Woutersen, R. A., Cassee, F. R. & van Bladeren, P. J. *Mutation Res.* **259**(3-4), 363-385 (1991).

S24. Xie, M. Z., *et. al.* Aldehydes with high and low toxicities inactivate cells by damaging distinct cellular targets. *Mut Res./Fund Molec Mech Mutagen.* **786**, 41-51 (2016).

S25. LoPachin, R.M. & Gavin, T. Molecular mechanisms of aldehyde toxicity: A chemical perspective *Chem Res Toxicol.* **27**, 1081-91 (2014). dx.doi.org/10.1021/tx5001046

S26. Songur, A., *et. al.* The effects of inhaled formaldehyde on oxidant and antioxidant systems of rat cerebellum during the postnatal development process. *Toxicology Mechanisms and Methods.* **18**(7) (2008). http://dx.doi.org/10.1080/15376510701555288

S27. Matveychuk, D., Dursun, S. M., Wood, P. L. & Baker G. B. Reactive aldehydes and neurodegenerative disorders. *Bull Clin Psychopharm* **21**(4), 277-288 (2011).

S28. Fitzmaurice, A. G., *et. al.* Aldehyde dehydrogenase inhibition as a pathogenic mechanism in Parkinson *Disease. Proc Natl Acad Sci.* **110**(2), 636–41 (2013).

S29. Vaglini, F., *et al.* Involvement of cytochrome P450 2E1 in the 1-methyl-4-phenyl-1,2,3,6-tetrahydropyridine-induced mouse model of Parkinson's disease. *J Neurochem.* **91**(2), 285–98 (2004).

S30. Jurnak, F. The Pivotal Role of Aldehyde Toxicity in Autism Spectrum Disorder: The Therapeutic Potential of Micronutrient Supplementation. *Nutr Metabolic Insights*. **8**(S1), 57-77 (2015). doi:10.4137/NMI.S29531.

S31. Leikauf, G. D. United States Environmental Protection Agency (EPA) final report: Inhalation of aldehydes and effects of breathing. EPA Grant Number R828112CO49.

S32. Venkata, R. P. & Subramanyam, R. Evaluation of the deleterious health effects of consumption of repeatedly heated vegetable oil. *Toxicol Rep.* **3**, 636-643 (2016).

S33. Osim, E. E., Owu, D. U. & Etta K. M. Arterial pressure and lipid profile in rats following chronic ingestion of palm oil diets. *Afr J Med Sci.* **25**, 335-40 (1996).

S34. Leong, X. F., Aishah, A., Nor Aini, U., Das S. & Jaarin K. Heated palm oil causes rise in blood pressure and cardiac changes in heart muscle in experimental rats. *Arch Med Med Res.* **39**, 567-72 (2008).

S35. Owu, D. U., Orie, N. N. & Osim, E. E. Altered responses of isolated aortic smooth muscle following chronic ingestion of palm oil diets in rats. *Afr J Med Med Sci.* **26**, 83-86 (1997).

S36. Owu, D. U., Osim, E. E. & Ebong, P. E. Serum liver enzymes profile of Wistar rats following chronic consumption of fresh or oxidized palm oil diets. *Acta Trop.* **69**, 65-73 (1998).

S37. Battino, M. *et al.* Feeding fried oil changes antioxidant and fatty acid pattern of rat and affects rat liver mitochondrial respiratory chain components. *J. Bioenergetics Biomembranes* **34**(2), 127-134 (2002).

S38. Quiles, J. L. *et al.* The intake of fried virgin olive or sunflower oils differentially induces oxidative stress in rat liver microsomes. *Brit. J. Nutr.* **88**, 57-65 (2002).

S39. Gliszczyńska-Świgłol, A., Sikorska, E., Khmelinskii, I. & Sikorski, M. Tocopherol content in edible plant oils. *Pol. J. Food Nutr.* Sci. **57**(4A): 157-161 (2007).

S40. Sprince, H., Parker, C. M., Smith, G. G. & Gonzales L. J. Protective action of ascorbic acid and sulfur compounds against acetaldehyde toxicity: implications in alcoholism and smoking. *Agents Actions* **5**, 164–173 (1975).

S41. Salaspuro, V., *et al.* (2002) Removal of acetaldehyde from saliva by a slow-release buccal tablet of L-cysteine. *Int J Cancer* **97**, 361–364 (2002).

S42. Salaspuro, V. J., Hietala, J. M., Marvola, M. L. & Salaspuro, M. P. Eliminating carcinogenic acetaldehyde by cysteine from saliva during smoking. *Cancer Epidemiol Biomarkers Prev* **15**, 146–49 (2006).

S43. Linderborg, K. *et al.* Reducing carcinogenic acetaldehyde exposure in the achlorhydric stomach with cysteine. *Alcohol Clin Exp Res*, **35**(3), 1-7 (2011). DOI: 10.1111/j.1530-0277.2010.01368.x

S44. Lienhard, G. E & Jencks, W. P. Thiol addition to the carbonyl group. Equilibria and kinetics. *J Am Chem Soc.* **88**(17), 3982-95 (1966).

S45. Regulska-Ilow, B., Ilow, R., Biernat, J., Grajeta, H., & Kowalski, P. The influence of bioflavonoids from the radix of Scutellaria baicalensis on liver function of laboratory rats fed with fresh and oxidized fats. *Adv Clin Exp Med.* **16**(1), 13-20 (2007).

S46. Regulska-Ilow, B., Biernat, J., Grajeta, H.M, Ilow, R. & Drzewicka, M. Influence of bioflavonoids from the radix extract of *Scutellaria baicalensis* on the level of serum lipids, and the development of laboratory rats fed with fresh and oxidized fats. *Molec. Nutr. Food. Res.* **48**(2), 123-128 (2004).

S47. Willett, W.C. & Ascherio, A. Trans-fatty acids: Are the effects only marginal? *Am. J. Pub. Health* **84**, 722-724 (1994).

S48. Guillod, T. Interpolations, courbes de Bézier et B-splines. *Bulletin de la société des enseignants neuchatelois de sciences*. **34**, 1-50 (2008).

Supplementary Materials Section Table and Table Legend

**Aldehyde Classification**	**PC1**	**PC2**
trans-2-Alkenals	**0.71**	**0.65**
trans,trans-Alka-2,4-dienals	**0.89**	0.38
4,5-Epoxy-*trans*-2-alkenals	**0.84**	0.45
4-Hydroperoxy/Hydroxy-*trans*-2-alkenals	**0.83**	0.52
cis,trans-Alka-2,4-dienals	**0.85**	0.48
n-Alkanals	**0.77**	0.60
cis-2-Alkenals	0.42	**0.90**

Table S1. Factor loadings vectors after Varimax rotation for ^{1}H NMR-detectable aldehydic LOP classes obtained from principal component analysis (PCA) of their concentrations generated in culinary frying oils when heated according to LSSFEs.

Supplementary Materials Section Figures and Figure Legends

Figure S1

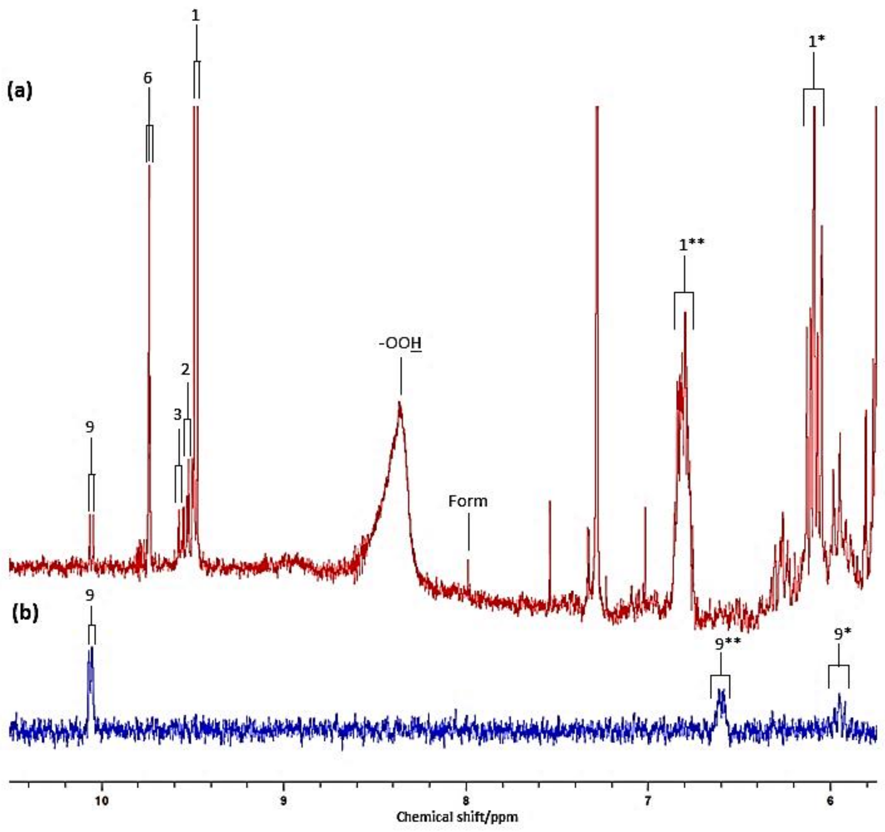

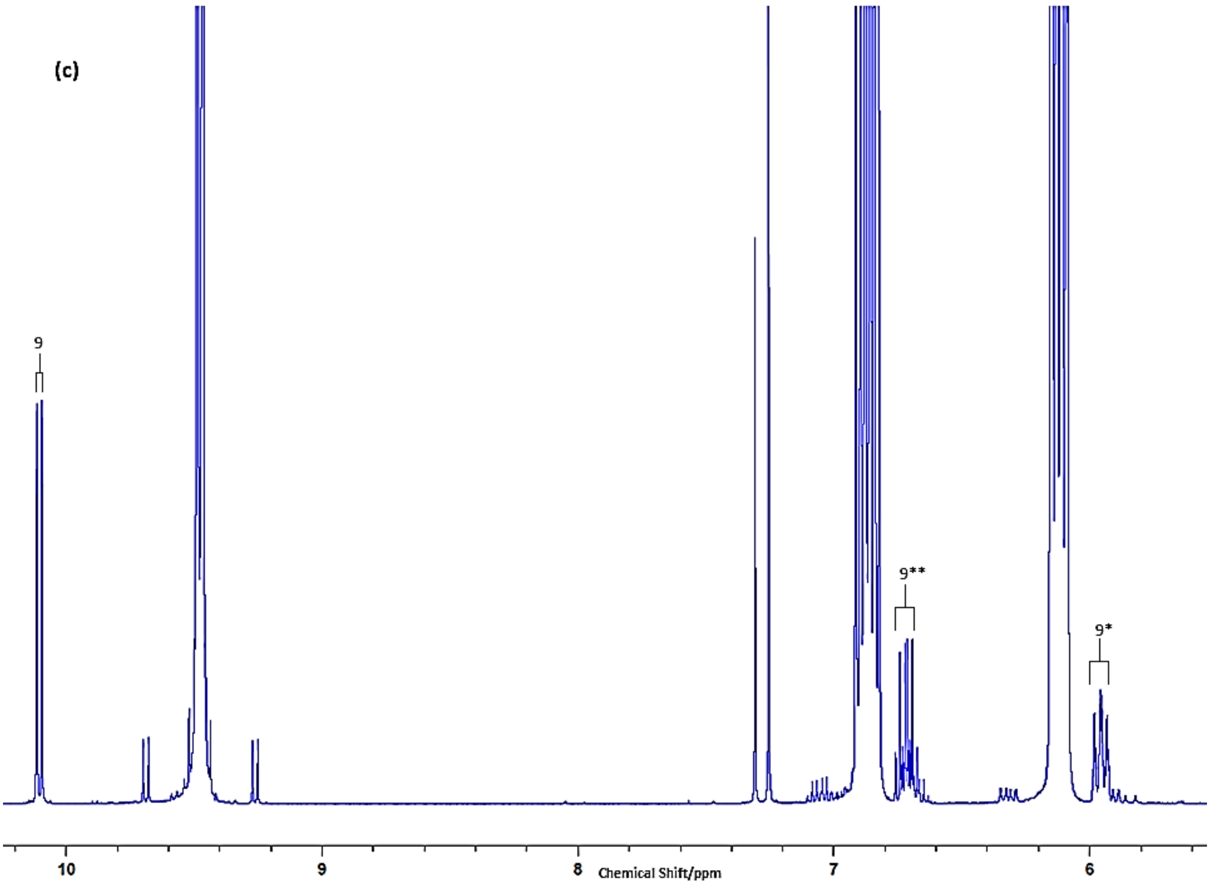

Figure S1. (a) and **(b)**. Partial single-pulse ^{1}H and corresponding one-dimensional (1D) ^{1}H-^{1}H TOCSY NMR spectra, respectively, of the MRAFO product thermally-stressed according to LSSFEs at 180°C for a period of 90 min., confirming clear connectivities between the 10.07 ppm aldehydic function signal (*d*, *j* = 8.1 Hz) and vinylic proton resonances centred at 5.97 (*m*) and 6.70 ppm (*m*). This spectral profile is fully consistent with those of *cis*-2-alkenals, e.g. that of *cis*-butenal (*cis*-crotonaldehyde) shown in **(c)**, which is the corresponding ^{1}H NMR profile of a commercial sample of butenal (crotonaldehyde) obtained from the manufacturer as a 20:1 molar ratio of its *trans-(E-)*:*cis-(Z-)* isomers. Abbreviations: 9, 9* and 9**, aldehydic-C<u>H</u>O, and vinylic 2(α)-C<u>H</u> and 3(β)-C<u>H</u> function ^{1}H NMR resonances, respectively, of *cis*-2-alkenals [*cis*-2-butenal in (c)]; 1, 2, 3 and 6, aldehydic-C<u>H</u>O function signals of *trans*-2-alkenals, *trans,trans*-alka-2,4-dienals; 4,5-epoxy-*trans*-2-alkenals and *n*-alkanals respectively (as in Figure 1); 1* and 1**, vinylic 2(α)-C<u>H</u> and 3(β)-C<u>H</u> function ^{1}H NMR resonances, respectively, of *trans*-2-alkenals; -OOH, broad hydroperoxy function –OO<u>H</u> proton resonance, predominantly that of HPMs; Form, formic acid arising from the oxidation of the aldehydic LOP formaldehyde. Resonances of much higher intensity located at δ = 9.48 (*d*) ppm, 6.84 (*dq*) and 6.11 ppm (*ddq*) arise from the -C<u>H</u>O function, 3(β)-position vinylic and 2(α)-position vinylic protons, respectively, of *trans*-2-butenal.

Figure S2

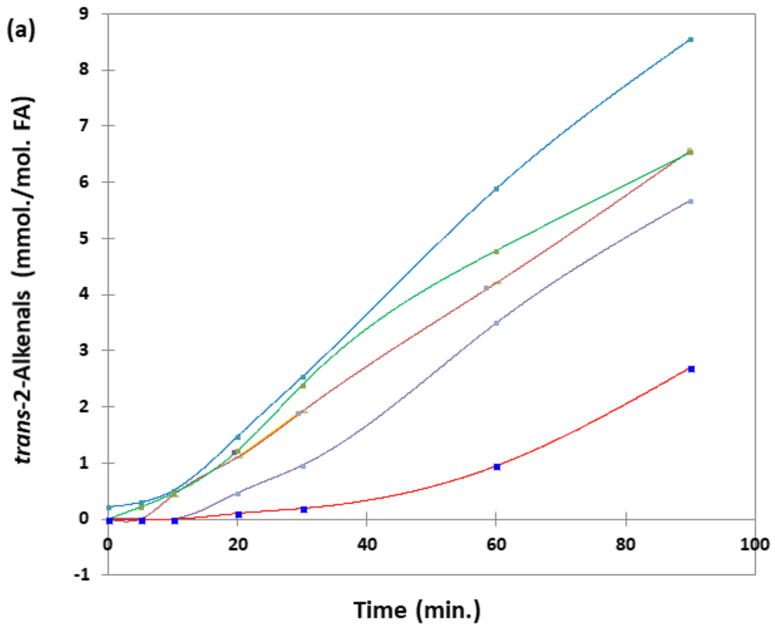

(a)
trans-2-Alkenals (mmol./mol. FA)
Time (min.)
9
8
7
6
5
4
3
2
1
0
-1
0
20
40
60
80
100

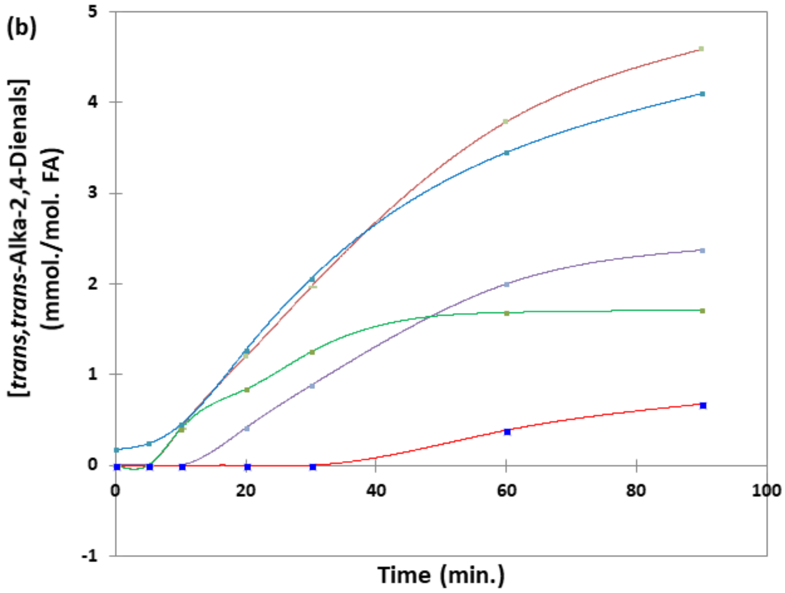
(b)
[*trans,trans*-Alka-2,4-Dienals]
(mmol./mol. FA)
5
4
3
2
1
0
-1
0
20
40
60
80
100
Time (min.)

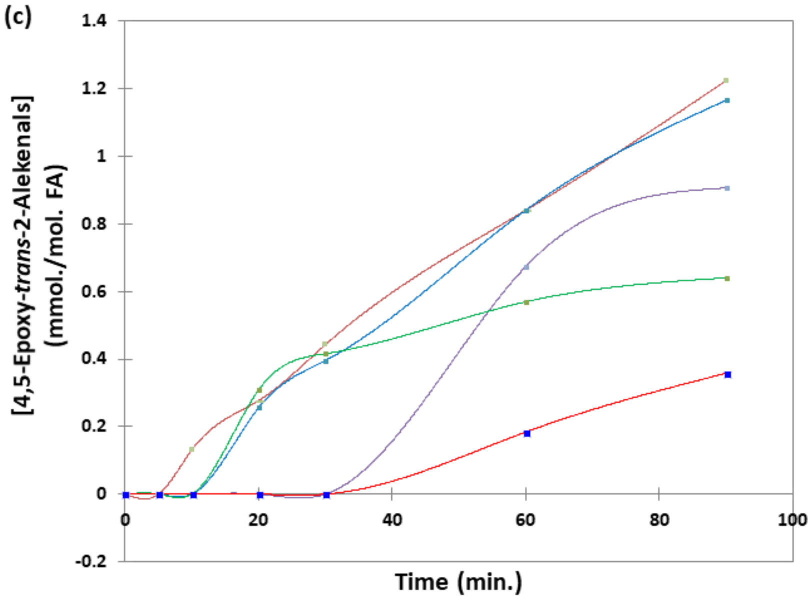
(c)
[4,5-Epoxy-*trans*-2-Alekenals]
(mmol./mol. FA)
1.4
1.2
1
0.8
0.6
0.4
0.2
0
-0.2
0
20
40
60
80
100
Time (min.)

(d)

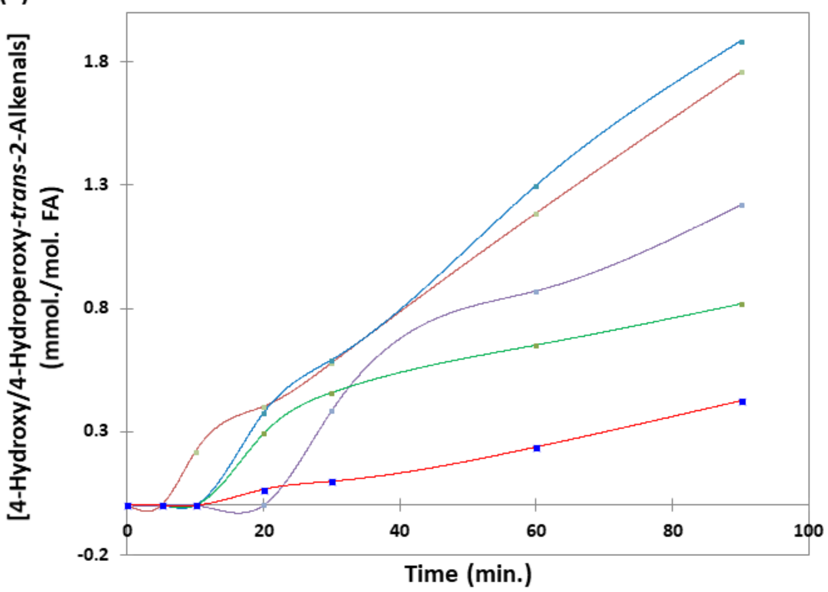
[4-Hydroxy/4-Hydroperoxy-*trans*-2-Alkenals] (mmol./mol. FA)
1.8
1.3
0.8
0.3
-0.2
0
20
40
60
80
100
Time (min.)

(e)

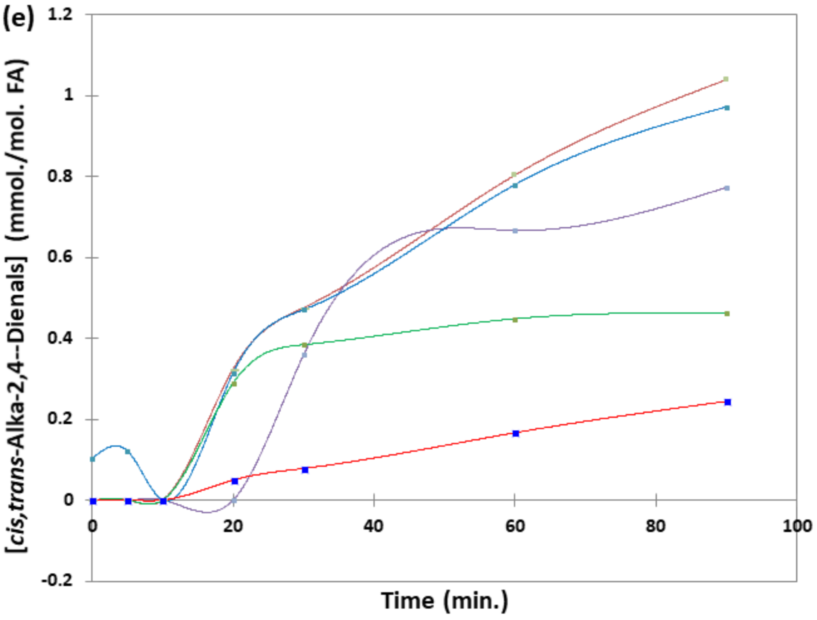
[*cis,trans*-Alka-2,4--Dienals] (mmol./mol. FA)
1.2
1
0.8
0.6
0.4
0.2
0
-0.2
0
20
40
60
80
100
Time (min.)

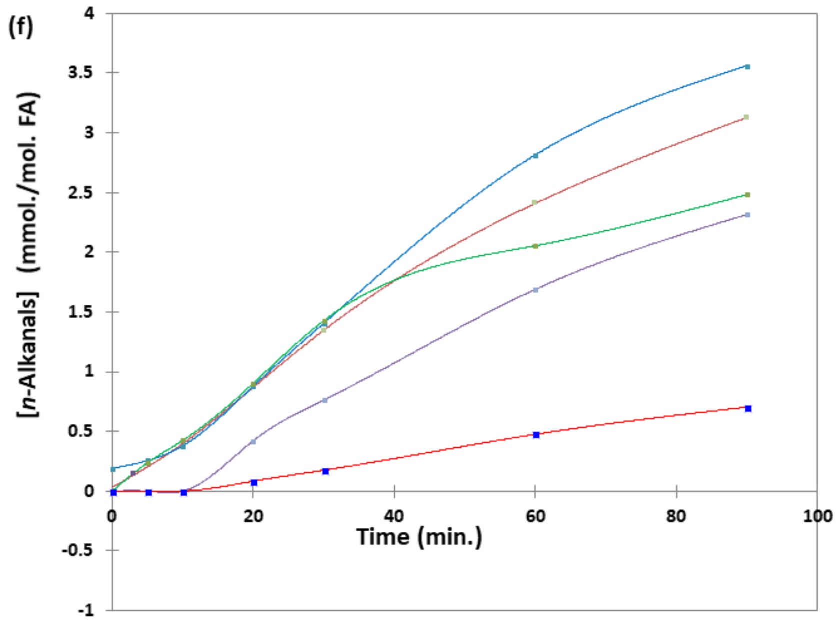
(f)
[n-Alkanals] (mmol./mol. FA)
Time (min.)
4
3.5
3
2.5
2
1.5
1
0.5
0
-0.5
-1
0
20
40
60
80
100

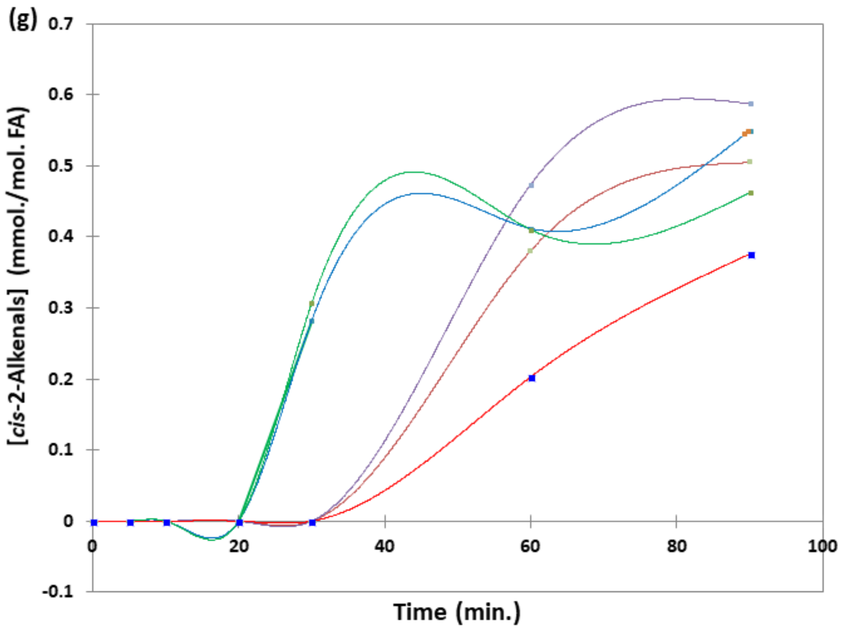
(g)
[cis-2-Alkenals] (mmol./mol. FA)
Time (min.)
0.7
0.6
0.5
0.4
0.3
0.2
0.1
0
-0.1
0
20
40
60
80
100

Figure S2. Cubic spline polynomial plots of mean total ^{1}H NMR-determined **(a)** *trans*-2-alkenal, **(b)** *trans*,*trans*-alka-2,4-dienal, **(c)** 4,5-epoxy-*trans*-2-alkenals; **(d)** combined 4-hydroxy/4-hydroperoxy-*trans*-2-alkenals; **(e)** *cis,trans*-alka-2,4-dienals; **(f)** *n*-alkanals; and **(g)** *cis*-2-alkenals (mmol./mol. FA) as a function of LSSFE heating time for sunflower (blue curves), corn (brown), extra virgin olive (green), canola (mauve) and MRAFO oils (red). The cubic spline analysis performed on our time-dependent aldehyde concentration datasets involved a piecewise function of third-degree polynomials[S48].

Figure S3

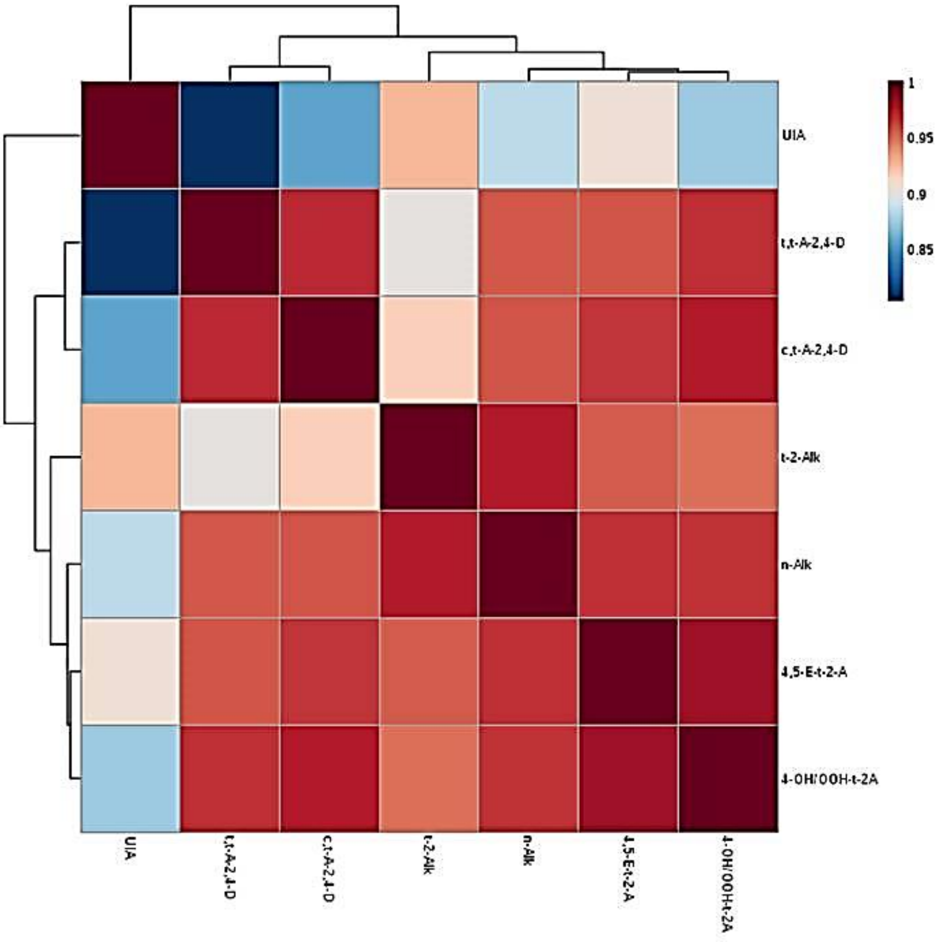

Figure S3. Correlation feature diagram showing correlations between the cube root-transformed and Pareto-scaled intensities of -CHO function resonance ISBs assigned to *n*-alkanals (n-Alk), *trans*-2-alkenals (t-2Alk), *trans*,*trans*- and *cis*,*trans*-alka-2,4-dienals (t,t-A-2,4-D and c,t-A-2,4-D respectively), 4,5-epoxy-*trans*-2-alkenals (4,5-E-t-2A), a combination of 4-hydroxy- and 4-hydroperoxy-*trans*-2-alkenals (4-OH/OOH-t-2A), and the previously unidentified aldehydic LOP class comprising *cis*-2-alkenals (UIA) in all culinary oil samples thermally-stressed according to LLSFEs at 180ºC for periods of 60-90 min. Pearson correlation coefficient (r) values ranged from 0.86 to 0.98 (colour codings for these are provided in the top right-hand corner panel). The strongest correlations were those between *trans*-2-alkenals and *n*-alkanals; *trans*,*trans*-alka-2,4-dienals, *cis*,*trans*-alka-2,4-dienals and 4-hydroxy/4-hydroperoxy-*trans*-2-alkenals; 4,5-epoxy-*trans*-2-alkenals and 4-hydroxy/4-hydroperoxy-*trans*-2-alkenals. The *cis*-2-alkenal (UIA) resonance ISB had somewhat weaker correlations between those observed for each of the other aldehydes; however, the strongest one was that with *trans*-2-alkenals (r = 0.93), which suggests that it may arise as a thermal isomerism product of this aldehyde class.

Figure S4

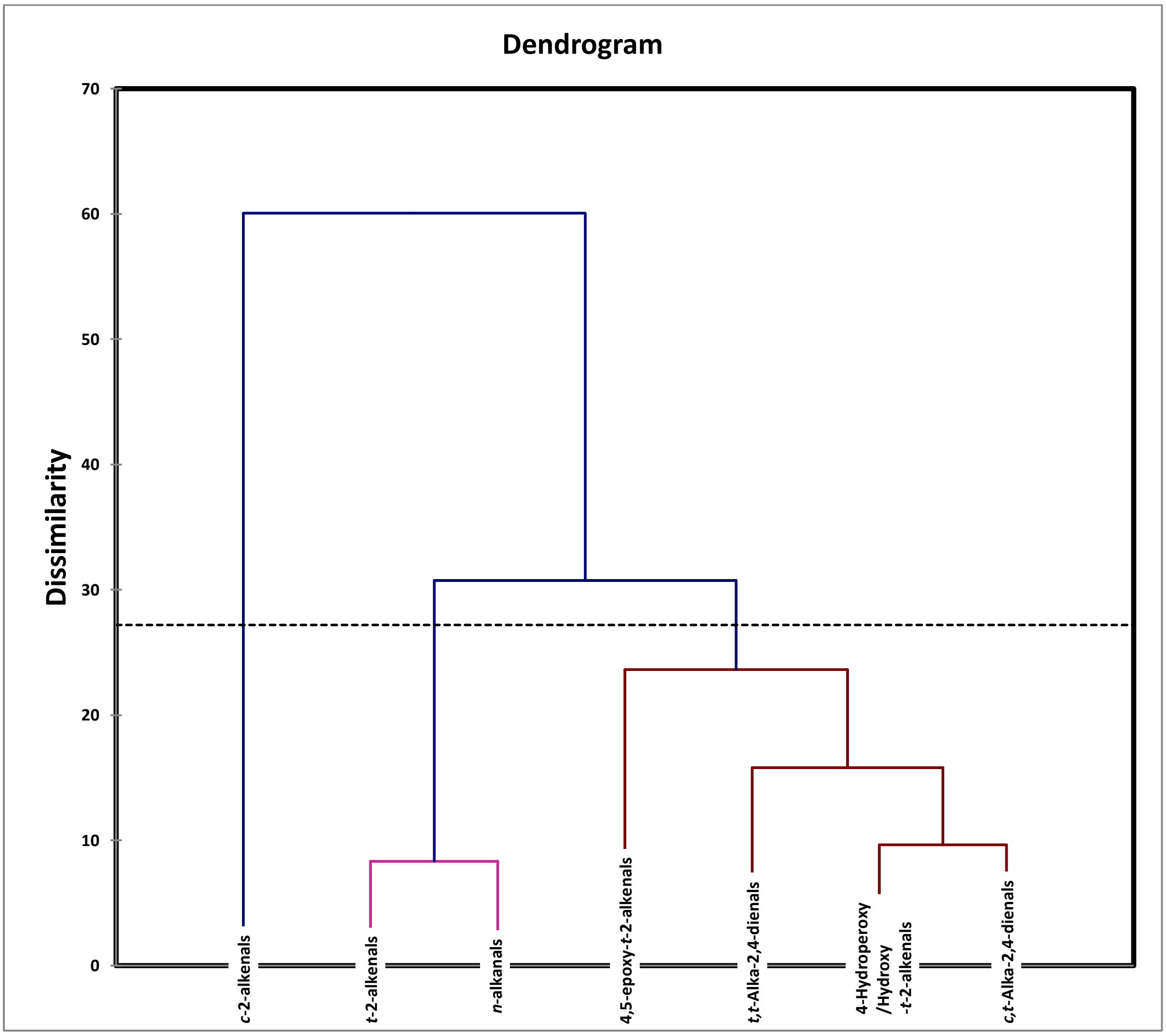

Figure S4. Agglomerative hierarchical clustering (AHC) analysis dendogram of ^{1}H NMR-detectable aldehyde classes generated in culinary oils heated according to LSSFEs (0-90 min. at 180ºC). Data from all five oils and all seven time-points investigated were included in this multivariate analysis strategy, and FA-normalised aldehyde concentrations were glog-transformed and Pareto-scaled prior to analysis. The different colourations indicate 3 distinct clusters for the 7 ^{1}H NMR-detectable classes of aldehydes monitored.
